# ABL1-mediated phosphorylation promotes FOXM1-related tumorigenicity by Increasing FOXM1 stability

**DOI:** 10.1038/s41418-024-01339-w

**Published:** 2024-07-26

**Authors:** Qincai Dong, Di Wang, Caiwei Song, Chunxue Gong, Yue Liu, Xinwei Zhou, Junjie Yue, Yong Hu, Hainan Liu, Lin Zhu, Xiayang Niu, Tong Zheng, Xun Zhang, Jing Jin, Tingting Wang, Ruixia Ju, Chen Wang, Qian Jiang, Ting Gao, Yanwen Jin, Ping Li, Yan Wang, Chunmei Zhang, Guang-Fei Wang, Cheng Cao, Xuan Liu

**Affiliations:** 1 State Key Laboratory of Pathogen and Biosecurity, Beijing Institute of Biotechnology, 100850 Beijing, China; 2 Institute of Health Sciences, Anhui University, Hefei, 230601 China; 3 Clinical Biobank Center, Medical Innovation Research Division, Chinese PLA General Hospital, 100853 Beijing, China; 4 Shanghai Key Laboratory of Regulatory Biology, Institute of Biomedical Sciences and School of Life Sciences, East China Normal University, Shanghai, 200241 China

**Keywords:** Cell biology, Molecular biology

## Abstract

The transcription factor FOXM1, which plays critical roles in cell cycle progression and tumorigenesis, is highly expressed in rapidly proliferating cells and various tumor tissues, and high FOXM1 expression is related to a poor prognosis. However, the mechanism responsible for FOXM1 dysregulation is not fully understood. Here, we show that ABL1, a nonreceptor tyrosine kinase, contributes to the high expression of FOXM1 and FOXM1-dependent tumor development. Mechanistically, ABL1 directly binds FOXM1 and mediates FOXM1 phosphorylation at multiple tyrosine (Y) residues. Among these phospho-Y sites, pY575 is indispensable for FOXM1 stability as phosphorylation at this site protects FOXM1 from ubiquitin-proteasomal degradation. The interaction of FOXM1 with CDH1, a coactivator of the E3 ubiquitin ligase anaphase-promoting complex/cyclosome (APC/C), which is responsible for FOXM1 degradation, is significantly inhibited by Y575 phosphorylation. The phospho-deficient FOXM1(Y575F) mutant exhibited increased ubiquitination, a shortened half-life, and consequently a substantially decreased abundance. Compared to wild-type cells, a homozygous Cr-Y575F cell line expressing endogenous FOXM1(Y575F) that was generated by CRISPR/Cas9 showed obviously delayed mitosis progression, impeded colony formation and inhibited xenotransplanted tumor growth. Overall, our study demonstrates that ABL1 kinase is involved in high FOXM1 expression, providing clear evidence that ABL1 may act as a therapeutic target for the treatment of tumors with high FOXM1 expression.

## Introduction

FOXM1, belonging to the Forkhead family of transcription factors, plays an important role in diverse biological processes, including cell cycle progression [1–3], tumorigenesis [4, 5], differentiation [6], inflammation [7], organ regeneration [8] and drug resistance [9, 10]. Among these functions, its role in cell cycle regulation is fundamental and has been well studied. A cluster of cell cycle-related genes are targeted by FOXM1, which regulates G1/S and G2/M transition [11–13], the progression of S and mitotic phases, and the timely exit of the cell cycle. During the G1/S transition, FOXM1 regulates transcription of the Skp1-Cullin 1-F-box (SCF) ubiquitin ligase subunits *SKP2* and *CKS1*, which target the cyclin-dependent kinase inhibitor (CDKI) proteins P21 and P27 for degradation, thereby promoting S phase entry [14]. More reports emphasize the function of FOXM1 during the G2/M transition and mitosis. In late G2 phase, FOXM1 ensures proper mitotic entry by controlling the transcription of a cluster of genes critical for the G2/M transition, such as *CYCLIN B1, AURORA B, PLK1, CENP F and CDC25B* [2, 13]. Accordingly, loss of FOXM1 leads to mitotic entry delay, spindle defects, chromosome mis-segregation and polyploidization [3]. In agreement with its function, the expression level of FOXM1 changes periodically along with cell cycle progression. FOXM1 is poorly detectable at G0 and early G1 phases and increases in S phase. The FOXM1 level peaks in G2 phase, is sustained throughout mitosis, and eventually decreases for mitotic exit at the end of M phase through ubiquitin‒proteasome degradation [1, 15].

FOXM1 is commonly expressed in embryonic tissues and adult tissues composed of actively dividing cells, such as the testis, thymus and intestine [16], indicative of its pro-proliferative effects and potential correlation with oncogenesis. Indeed, the high expression of FOXM1 in numerous cancer cell lines and a variety of tumors is always associated with tumor initiation [17], development [18], invasion [19, 20], metastases [5, 7, 21–23] and poor prognosis [24–26] by directly potentiating the expression of tumor-related genes, such as *ERα* [27], *TOPO-2α* [28], *c-Myc* [17], *DLX1* [19] and *PDGF-A* [29]. FOXM1-depleted mice display significantly compromised tumor growth and metastasis [7], whereas FOXM1 overexpression contributes to poor prognosis in patients with lung adenocarcinomas, meningioma and acute lymphoblastic leukemia. Preventing FOXM1 overexpression represents a novel therapeutic strategy against tumors with high FOXM1 expression [25, 30].

ABL nonreceptor tyrosine kinase, which was first identified as an oncogene product (e.g., BCR-ABL) generated by chromosomal translocation in chronic myelogenous leukemia (CML), is ubiquitously expressed in mammalian cells and is also known as cellular ABL (e.g., ABL1), which plays essential physiological roles in multiple biological processes, including cell proliferation [31, 32], differentiation [33], tumorigenesis [34], stress responses [35, 36], cell migration and adhesion [37]. The N-terminus of ABL1 contains Src homology 3 (SH3), SH2, and tyrosine kinase domains, while the C-terminus has three independent nuclear localization signals (NLSs), allowing it to shuttle between the nucleus and cytoplasm. The ABL1-related gene ABL2, which is the other member of the ABL kinase family, exhibits functional redundancy with ABL1. Mice deficient in both *abl1* and *abl2* die during the embryonic period and suffer from defects in neurulation; most *abl1*^*−/−*^ mice die 1 to 2 weeks after birth, suggesting that ABL1 and ABL2 play critical roles in mouse development [38, 39]. Recent studies have demonstrated the overexpression or dysregulated activation of ABL1 in cancers such as lung, breast, colon, and renal carcinoma, indicative of its potential role in solid tumors [34, 36, 40]. ABL1 kinase inhibitors such as imatinib and nilotinib, which have been approved for the treatment of BCR-ABL-positive CML, have been evaluated in solid tumor treatment.

The transcriptional activity and stability of FOXM1 are mainly regulated by Ser/Thr phosphorylation [2, 41], whereas its tyrosine phosphorylation is seldom reported. Here, we show that ABL1 kinase-mediated FOXM1 tyrosine phosphorylation at Y575 is indispensable for FOXM1 stability in the cell. CRISPR-modified cells expressing FOXM1(Y575F), a tyrosine phosphorylation-deficient mutant, exhibited impaired mitosis and compromised tumorigenesis compared to wild-type cells. Our study highlights an essential role for ABL1 kinase in FOXM1 phosphorylation and stability, which may offer a strategy for the treatment of tumors with FOXM1 overexpression.

## Results

### FOXM1 expression is posttranscriptionally regulated by ABL1 kinase

**Fig. 1:**
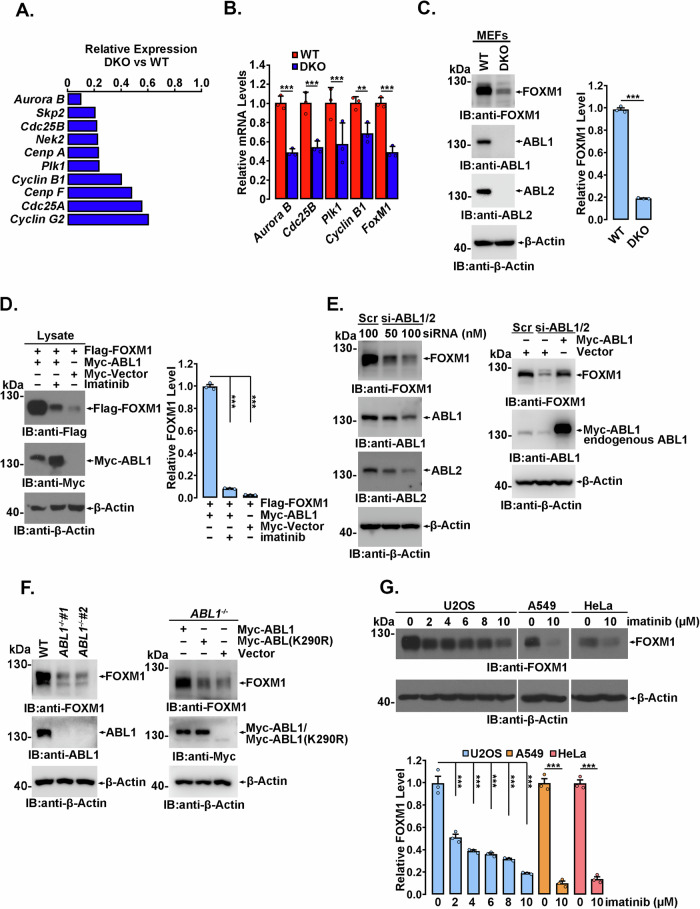
ABL1 regulates FOXM1 expression. **A** FOXM1 target genes that were downregulated by *abl1/abl2* double knockout were revealed by wild-type and *abl1*^*−/−*^*abl2*^*−/−*^ MEF transcriptome analysis and comparison. **B** Total RNA was extracted from wild-type and *abl1*^*−/−*^*abl2*^*−/−*^ MEFs, and the indicated RNA levels were determined by qRT‒PCR. Data shown represent the means ± SD of biological triplicates. **p < 0.01, ***p < 0.001, Student’s *t* test. **C** Lysates of wild-type and *abl1*^*−/−*^*abl2*^*−/−*^ MEFs were analyzed by immunoblotting, and the relative FoxM1 protein levels were quantified by ImageJ software and plotted, and the data are presented as the mean±S.D. of three independent analyses. ***p < 0.001, Student’s *t* test. **D** 293FT cells transfected with the indicated plasmids and treated with or without 10 µM imatinib for 18 h were analyzed by immunoblotting, and the relative expression of FOXM1 was quantified and statistically analyzed, and represented as mean ± S.D. of three independent analysis. ***p < 0.001, ANOVA. **E** HeLa cells were transfected with the indicated concentration of ABL1/ABL2 siRNA or scramble siRNA (as a control) for 72 h and were analyzed by immunoblotting (left). HeLa cells transfected with ABL1/ABL2 siRNA were infected with siRNA-resistant ABL1 lentivirus (MOI = 10) and analyzed by immunoblotting (right). **F** Lysates prepared from the indicated HeLa cells infected with (right) or without (left) the lentivirus expressing the indicated proteins were detected by immunoblotting. **G** The indicated cells treated with imatinib at different concentrations were analyzed by immunoblotting, and the relative expression of FOXM1 was quantified and statistically analyzed, and represented as mean ± S.D. of three independent analysis. ***p < 0.001, ANOVA.

Our previous comparative transcriptome analysis between wild-type (WT) and *abl1/abl2* double knockout (DKO) mouse embryonic fibroblasts (MEFs) revealed a wide range of differentially expressed genes that are regulated by ABL1 kinase, among which several FOXM1-downstream genes, such as *Aurora B, Cyclin B1*, and *Plk1*, were significantly downregulated by *abl1/abl2* DKO (Fig. 1A). The changes in the mRNA levels of these genes in DKO MEFs were further confirmed by qRT‒PCR (Fig. 1B). Meanwhile, we noticed that the mRNA level of *FoxM1* itself was modestly decreased in DKO MEFs (Fig. 1B). Furthermore, the protein level of FOXM1 was detected in WT and *abl1*^*−/−*^*abl2*^*−/−*^ MEFs. Surprisingly, the FOXM1 protein level in *abl1*^*−/−*^*abl2*^*−/−*^ MEFs was significantly decreased compared to its mildly changed mRNA level (Fig. 1C). Considering that *FOXM1* gene transcription is subjected to positive autoregulation by itself [42], posttranscriptional regulation of FOXM1 by ABL1 kinase may exist. To substantiate this hypothesis, exogenous Flag-FOXM1 was coexpressed with or without Myc-ABL1, and a remarkable increase in Flag-FOXM1 expression was observed when coexpressed with Myc-ABL1, which could be significantly inhibited by the ABL1 kinase inhibitor imatinib (Fig. 1D). This finding indicated that posttranscriptional regulation may be involved in the low expression of FOXM1 in *abl1*^*−/−*^*abl2*^*−/−*^ MEFs.

We then sought to verify whether ABL1 could impact FOXM1 expression in tumor cell lines with FOXM1 overexpression. In agreement with the results in MEFs, a substantial decrease in FOXM1 levels was observed in HeLa cells transiently transfected with siRNA against *abl1/abl2* (Fig. 1E and Supplementary Fig. S1A, left), which could be rescued by exogenously expressed siRNA-resistant ABL1 kinase (Fig. 1E and Supplementary Fig. S1A, right). CRISPR/Cas9-mediated *ABL1* knockout in HeLa cells (Fig. 1F and Supplementary Fig. S1B, left) resulted in markedly decreased FOXM1 expression, which could be rescued by exogenous Myc-ABL1 but not by the kinase-dead mutant Myc-ABL1(K290R) (Fig. 1F and Supplementary Fig. S1B, right). Accordingly, the ABL1 kinase inhibitor imatinib induced a significant dose-dependent decrease in FOXM1 expression in U2OS cells (Fig. 1G). Similar results were also observed in imatinib-treated A549 and HeLa cells (Fig. 1G). Further, treatment with the ABL1 inhibitors nilotinib and asciminib also resulted in a dramatically reduction in the FOXM1 level (Supplementary Fig. S1C), accompanied by suppression of the autophosphorylation of ABL1 at Y412, which indicates ABL1 activation. However, the nucleoplasmic distribution of FOXM1 was not much affected by ABL1 (Supplementary Fig. S1D–G). Collectively, these findings suggested that the FOXM1 level is extensively regulated by ABL1 tyrosine kinase in different types of cells.

### ABL1 kinase interacts with FOXM1 directly

**Fig. 2:**
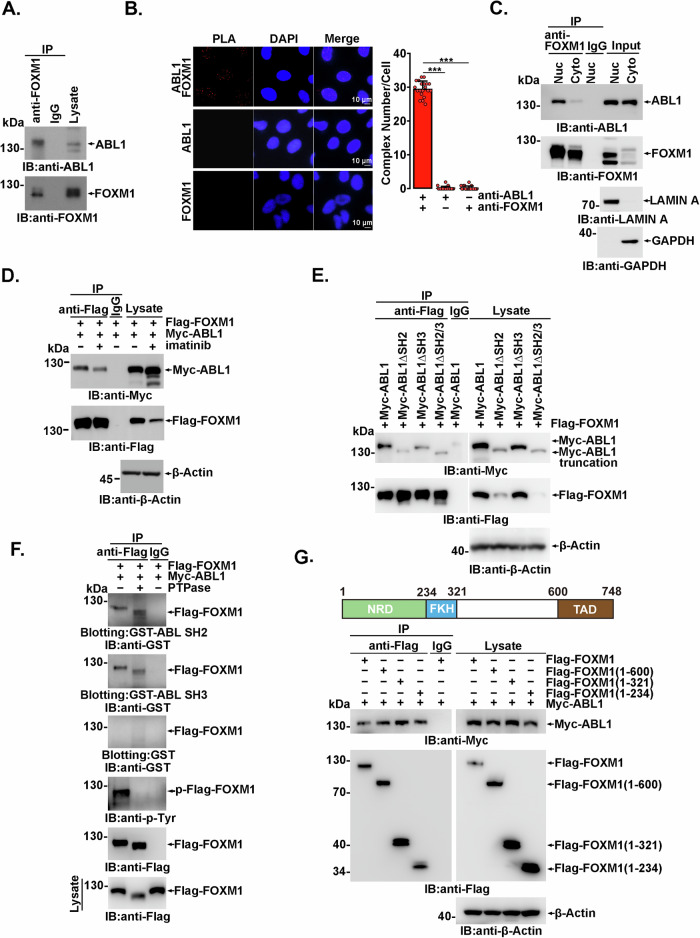
ABL1 interacts with FOXM1 directly. **A** Total lysates from U2OS cells were subjected to anti-FOXM1 or IgG (as a control) immunoprecipitation, and the immunoprecipitates were analyzed by immunoblotting. **B** U2OS cells were subjected to an in situ PLA assay with anti-ABL1 and anti-FOXM1 antibodies or one of them as a control. Red in situ PLA signals are displayed on the left and quantified on the right. Data shown represent the means ± SDs of biological triplicates. ***p < 0.001, Student’s *t* test. **C** Cytoplasmic or nuclear fractions from HeLa cells were subjected to anti-FOXM1 or IgG immunoprecipitation, and the immunoprecipitates were analyzed by immunoblotting. **D**, **E** 293FT cells transfected with the indicated plasmids were analyzed by immunoprecipitation and immunoblotting. **F** Lysates from 293FT cells coexpressing Flag-FOXM1 and Myc-ABL1 were treated with or without PTPase at 37 °C for 2 h and subjected to anti-Flag or IgG (as a control) immunoprecipitation, SDS‒PAGE separation and membrane transfer. Then, the PVDF membranes were incubated with soluble GST-ABL1 SH2, GST-ABL1 SH3 or GST (as a control) and immunoblotted with the indicated antibodies. **G** Schematic representations of FOXM1 (upper panel). 293FT cells transfected with the indicated plasmids were analyzed by immunoprecipitation and immunoblotting (lower panel).

Previous work has revealed that ABL kinase regulates the abundance of its partner protein by direct association and phosphorylation [43]. To further address ABL-dependent FOXM1 expression, the association between ABL1 and FOXM1 was investigated. The anti-FOXM1 immunoprecipitates prepared from cell lysates were analyzed by anti-ABL1 immunoblotting, and the presence of ABL1 in anti-FOXM1 immunoprecipitates was observed in both U2OS (Fig. 2A) and HeLa cells (Supplementary Fig. S2A). The in situ association of endogenous ABL1 and FOXM1 was further confirmed by proximity ligation assay (PLA) experiments. As shown in Fig. 2B, although FOXM1 mainly functions in the nucleus, signals from the FOXM1:ABL1 complex were detected in not only the nucleus but also the cytoplasm, which is consistent with the extensive cellular distribution of ABL1 kinase. The interaction between ABL1 and FOXM1 in the cytoplasm and nucleus was also demonstrated by coimmunoprecipitation in HeLa (Fig. 2C) and MCF-7 (Supplementary Fig. S2B) cells. ABL1 interacted with FOXM1 mainly in nucleus, and to a far less extent in cytoplasm. Next, lysates of 293FT cells coexpressing Flag-FOXM1 with Myc-ABL1 were subjected to immunoprecipitation and immunoblotting, and the association of exogenously expressed Flag-FOXM1 and Myc-ABL1 was confirmed (Fig. 2D and Supplementary Fig. S2C). The association between FOXM1 and ABL1 was not greatly affected by the ABL1 inhibitor imatinib, indicating that this association may not be dependent on ABL1 kinase activity (Fig. 2D and Supplementary Fig. S2C).

The N-terminal SH3 and SH2 domains of ABL1 kinase, which are responsible for substrate association, are highly conserved among Src family kinases. To further identify the FOXM1 binding region, an SH3 or SH2 deletion mutant was constructed. The immunoprecipitation results showed that deletion of one or both of the SH2 and SH3 domains significantly impaired, but did not completely eliminate, the association between ABL1 and FOXM1, indicating that other unidentified regions of ABL1 might also participate in FOXM1 association (Fig. 2E). Then, agarose bead-conjugated GST-ABL1 SH2, GST-ABL1 SH3 or GST-only proteins were incubated with the lysates of 293FT cells expressing Flag-FOXM1. A GST pull-down assay showed that both GST-ABL1 SH3 and GST-ABL1 SH2, but not GST alone, could bind Flag-FOXM1 in vitro (Supplementary Fig. S2D). To rule out indirect binding mediated by other components in the cell lysate, anti-Flag immunoprecipitates prepared from 293FT cells cotransfected with Flag-FOXM1 and Myc-ABL1 were treated with or without PTPase and then subjected to SDS‒PAGE and nitrocellulose membrane blotting. A slight decrease in FOXM1 mobility was observed with PTPase treatment (Fig. 2F). After incubation with eluates of GST-ABL1 SH3, GST-ABL1 SH2 or GST as a control, the membrane was immunoblotted with anti-GST antibody. The results showed direct binding between Flag-FOXM1 and GST-ABL1 SH3 or GST-ABL1 SH2 but not between Flag-FOXM1 and the GST protein, and binding was not greatly affected by PTPase treatment, indicating that this interaction may not depend on the phosphorylation status of FOXM1 (Fig. 2F).

Conversely, the region in FOXM1 that is required for association with ABL1 was also identified. Each functional domain of FOXM1 was constructed and coexpressed with Myc-ABL1. Compared to the other domains, the N-terminal repressor domain (NRD, aa 1-234) demonstrated a higher ABL1-binding capacity (Supplementary Fig. S2E), which was further confirmed by GST pull-down experiments (Supplementary Fig. S2F). Additionally, aa 322-600 of FOXM1 was also contributed to the association with Abl. However, unexpectedly, the expression of the FOXM1 transcriptional activation domain (TAD, aa 600-748) could not be detected for some unknown reason (Supplementary Fig. S2E). A FOXM1ΔTAD deletion mutant had little if any effect on the association with ABL, suggesting that the TAD might be dispensable for binding (Fig. 2G). Collectively, these findings demonstrate that ABL1 kinase directly associates with FOXM1 both in cells and in vitro.

### FOXM1 is phosphorylated by ABL kinase

**Fig. 3:**
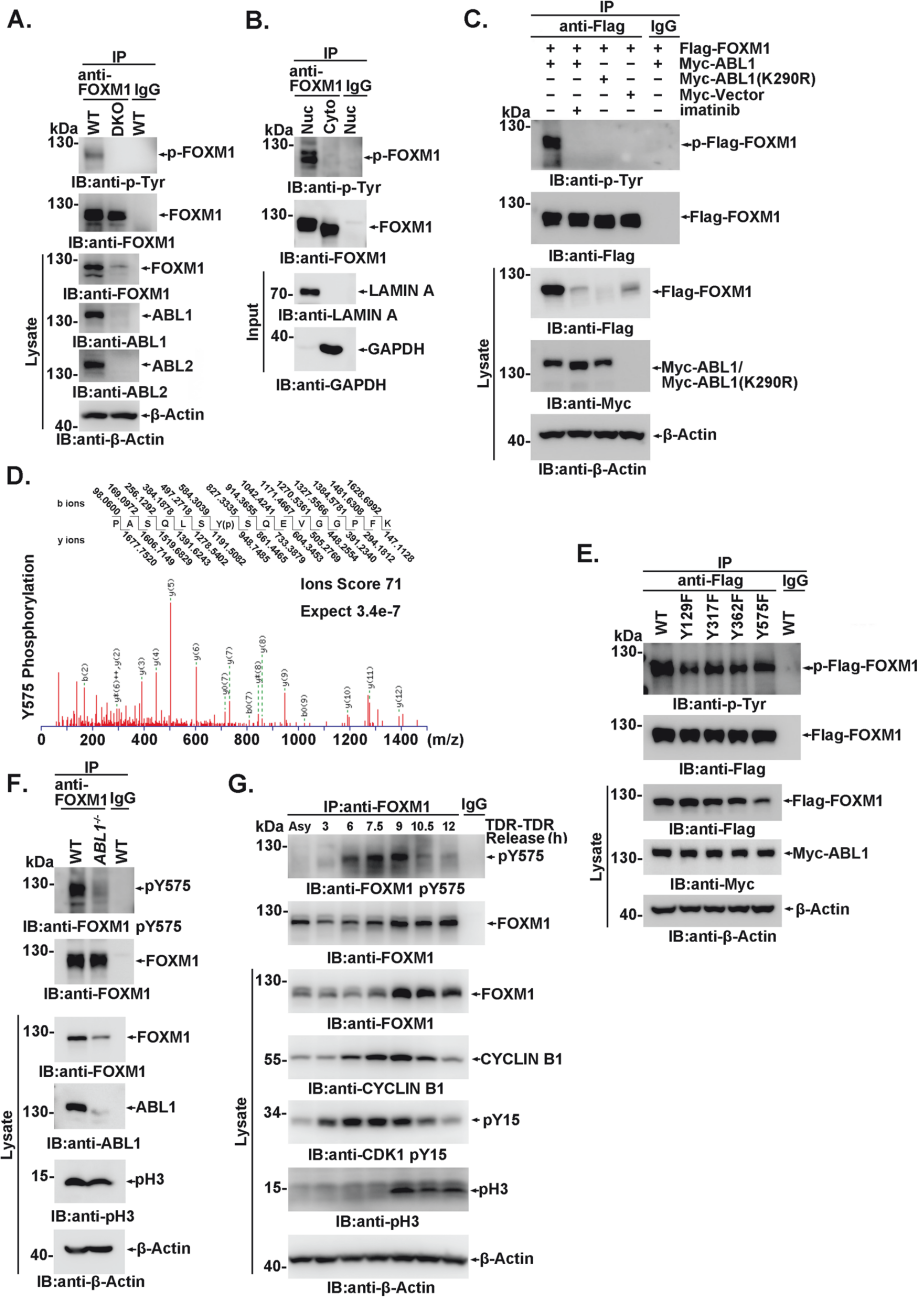
FOXM1 is phosphorylated by ABL1 mainly during the mitosis phase. **A** Lysates from wild-type and *abl1*^*−/−*^*abl2*^*−/−*^ MEFs were subjected to anti-FOXM1 or IgG (as a control) immunoprecipitation and analyzed by immunoblotting. **B** Cytoplasmic or nuclear fractions from HeLa cells were subjected to anti-FOXM1 or IgG immunoprecipitation, and the immunoprecipitates were analyzed by immunoblotting with the indicated antibodies. **C** 293FT cells transfected with the indicated plasmids were analyzed by immunoprecipitation and immunoblotting. **D** Anti-Flag immunoprecipitates prepared from the lysates of 293FT cells coexpressing Flag-FOXM1 and Myc-ABL1 were subjected to trypsinization and LC‒MS/MS analysis. Monophosphorylated peptides PASQLSY(p)SQEVGGPEF containing PO3-modified tyrosine residues were identified. **E** 293FT cells cotransfected Myc-ABL1 with the Flag-FOXM1WT or mutant plasmids were analyzed by immunoprecipitation and immunoblotting. **F** Wild-type or ABL1^*−/−*^ HeLa cells were harvested after double thymidine release for 9 h, then were subjected to anti-FOXM1 or IgG immunoprecipitation and analyzed by immunoblotting. **G** HeLa cells were arrested at the G1/S boundary by double thymidine block, released into fresh medium and harvested at the indicated time points. Lysates were subjected to anti-FOXM1 immunoprecipitation and immunoblotting analysis. In Fig. 3, all IP samples were balanced with the FOXM1 immunoprecipitation level by adjusting the loading volume, while the lysate was balanced with the β-Actin level.

The binding of FOXM1 with the ABL1 SH2 domain suggested that FOXM1 might be a substrate of ABL1 kinase. To verify this speculation, anti-FOXM1 immunoprecipitates were subjected to anti-p-Tyr immunoblotting. Endogenous FOXM1 phosphorylation of the tyrosine residue was observed, and this phosphorylation could be eliminated by *abl1/abl2* DKO (Fig. 3A) and by treatment with ABL1 inhibitors (Supplementary Fig. S3A, B). The tyrosine phosphorylated FOXM1 was distributed only in the nucleus but not in the cytoplasm in different type of cells (Fig. 3B and Supplementary Fig. S3C). The specificity of anti-p-Tyr immunoblotting was confirmed by phospho-L-tyrosine blocking (Supplementary Fig. S3D). Although imatinib-mediated kinase inhibition occurs instantly, an obvious decrease in FOXM1 tyrosine phosphorylation was observed at least one cell cycle duration (>12 h) after imatinib was administered (Supplementary Fig. S3E). Further experiments showed that exogenously expressed Flag-FOXM1 could be phosphorylated by Myc-ABL1 but not by kinase-dead Myc-ABL1(K290R) (Fig. 3C).

To investigate ABL1-mediated FOXM1 phosphorylation in detail, Flag-FOXM1 immunoprecipitates were analyzed by mass spectrometry. Four phosphotyrosine residues, pY129, pY317, pY362 and pY575, were identified (Fig. 3D and Supplementary Fig. S3F–H). Each Y to F mutation resulted in compromised phosphorylation compared to that of WT FOXM1 (Fig. 3E and Supplementary Fig. S3I), indicating the existence of multiple tyrosine phosphorylation sites on FOXM1. We noticed that the expression of the Flag-FOXM1(Y575F) mutant, but not other site mutants, was significantly downregulated in the lysates normalized by β-Actin (Fig. 3E), which provides insight into ABL1 kinase-regulated FOXM1 expression. To further confirm ABL1-mediated FOXM1 phosphorylation, especially at Y575, a FOXM1-pY575-specific polyclonal antibody was produced using the Cys-PASQLSY(p)SQEVGG peptide as an immunizing antigen (Supplementary Fig. S3J). Antibody specificity was verified by an in vitro kinase assay, in which the purified FOXM1 or FOXM1(Y575F) mutant was incubated with recombinant ABL1 kinase in the presence of ATP. Y575 site-specific phosphorylation of FOXM1 could be successfully detected by the FOXM1-pY575-specific antibody (Supplementary Fig. S3K). Moreover, anti-FOXM1-pY575 antibody immunoblotting was thoroughly blocked by the antigenic peptide (Supplementary Fig. S3L), indicating that the anti-FOXM1-pY575 antibody could specifically recognize Y575 phosphorylation. *ABL1* depletion significantly attenuated FOXM1 Y575 phosphorylation in HeLa cells, indicating that ABL1 is the major kinase responsible for FOXM1 Y575 phosphorylation (Fig. 3F).

Previous studies have reported that the phosphorylation status of FOXM1 is regulated by cell cycle progression[1, 12]. As a cell cycle-dependent kinase, ABL1 is activated in S phase and exhibits higher activity during mitosis [44], which may contribute to the tyrosine phosphorylation of FOXM1 in G2/M phase. To illustrate cell cycle-dependent FOXM1 tyrosine phosphorylation, cells were arrested at the G1/S boundary by double thymidine block and then released. G2/M phase was determined by CYCLIN B1 and phosphorylated histone H3 level. During early G2/M phase, the tyrosine phosphorylation of FOXM1 at Y575 was significantly increased, followed by the accumulation of FOXM1, which was delayed 1.5–2 h after Y575 phosphorylation, until late mitosis (Fig. 3G). Similar to the effect of double thymidine block, arrest of the cells in prometaphase by nocodazole block also significantly increased FOXM1 tyrosine phosphorylation during early mitosis (Supplementary Fig. S3M). The Ser/Thr phosphorylation of FOXM1 mediated by a number of kinases has been reported, but imatinib treatment had no significant effect on the Ser/Thr phosphorylation of FOXM1 (Supplementary Fig. S3N). Further, FOXM1 interacted with and was phosphorylated by ABL1 but not ABL2 (Supplementary Fig. S3O, P). These findings collectively indicated that ABL1-mediated tyrosine phosphorylation of FOXM1 mainly occurred during G2/M phase, which is consistent with and contributes to the role of FOXM1 in mitosis.

### FOXM1 is stabilized by ABL1 kinase-mediated Y575 phosphorylation

**Fig. 4:**
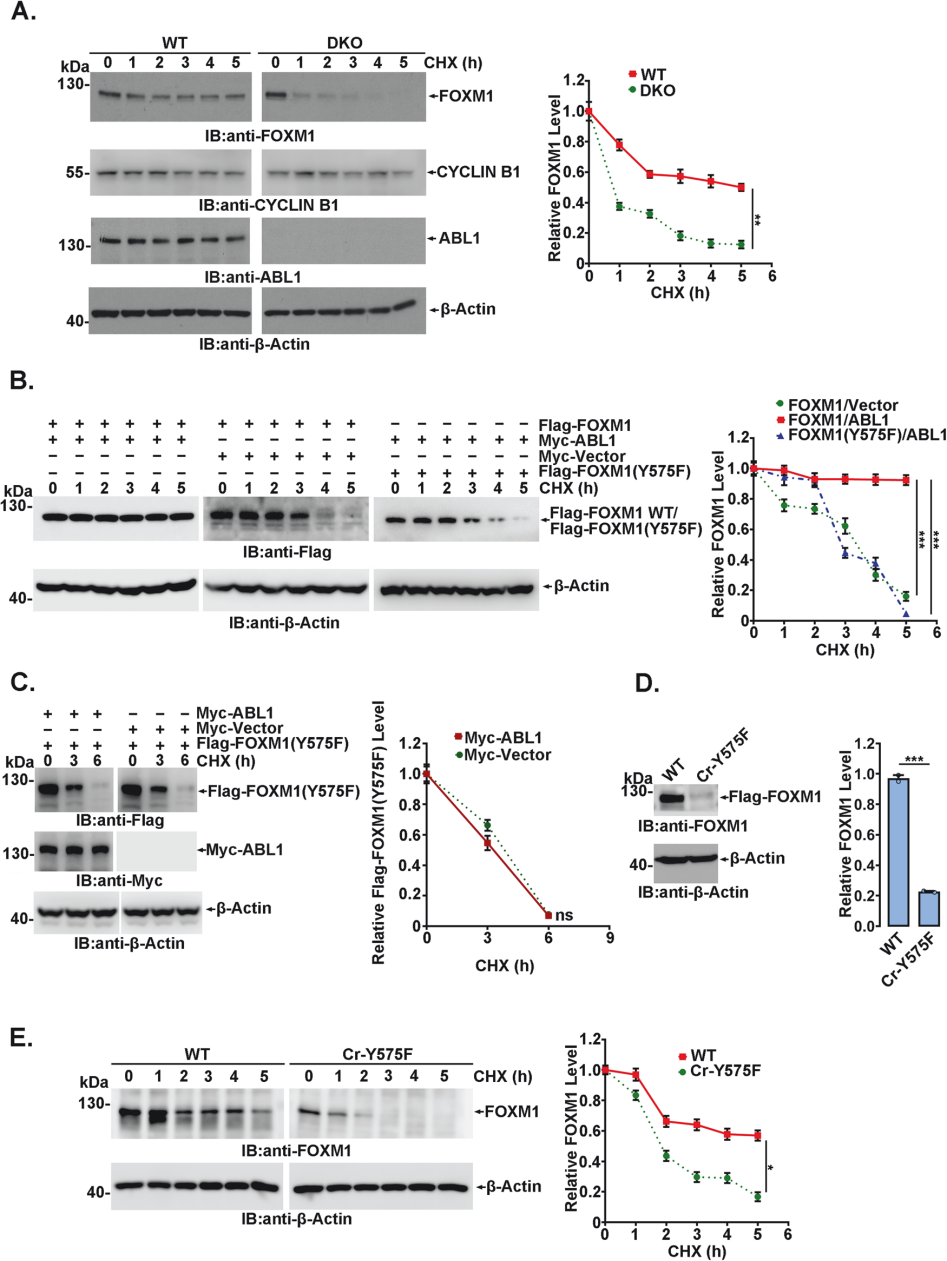
Y575 phosphorylation regulates FOXM1 protein stability. Wild-type and *abl1*^*−/−*^*abl2*^*−/−*^ MEFs (**A**) or 293FT cells cotransfected with the indicated plasmids (**B** and **C**) and treated with 100 µg/ml cycloheximide (CHX) for the indicated hours were harvested and analyzed by immunoblotting. The protein level was quantified by grayscale scanning by ImageJ software and plotted in the right panel. The data shown are the means ± SDs of three independent experiments. **p < 0.01, ***p < 0.001, Student’s *t* test. **D** FOXM1 levels in Cr-Y575F and wild-type cells were detected by immunoblot, and the relative protein levels were quantified by ImageJ software and plotted, and represented as mean ± S.D. of three independent analysis. ***p < 0.001, Student’s *t* test. **E** The stability of FOXM1 protein in Cr-Y575F and wild-type cells was analyzed as described in **A**. *p < 0.05, Student’s *t* test.

To assess whether FOXM1 stability is generally regulated by tyrosine phosphorylation, FOXM1 levels were detected using an asynchronized cell population at the indicated time points after cycloheximide (CHX) treatment to inhibit novel protein translation. In *abl1*^*−/−*^*abl2*^*−/−*^ MEFs, the cell cycle-independent half-life of FOXM1 was less than 1 h, which was significantly reduced compared with that in wild-type MEFs (Fig. 4A). In concert, the half-life of exogenously expressed Flag-FOXM1 WT was considerably prolonged by Myc-ABL1 coexpression (Fig. 4B). In contrast to Flag-FOXM1, Flag-FOXM1(Y575F) demonstrated significantly impaired stability even in the presence of Myc-ABL1, which was comparable to Flag-FOXM1 expressed alone (Fig. 4B). Notably, the stability of Flag-FOXM1(Y575F) was not strongly regulated by Myc-ABL1 coexpression (Fig. 4C), suggesting that the stability of FOXM1 was mainly regulated by ABL-mediated phosphorylation at the Y575 site.

To further confirm the critical role of Y575 phosphorylation in FOXM1 stability, CRISPR-mediated gene editing was used to introduce a mutation into FOXM1 genomic loci that resulted in the expression of a Y to F mutation at amino acid 575 in FOXM1. HeLa-derived Cr-Y575F cell line was established and verified by genomic sequencing for the mutation region, as shown in Supplementary Fig. S4A. As expected, Cr-Y575F cells demonstrated a significantly reduced FOXM1 level (Fig. 4D), and the stability of FOXM1(Y575F) was obviously decreased in Cr-Y575F cells compared to parental HeLa cells (Fig. 4E). In addition, FOXM1(Y575F) displayed a nucleoplasmic distribution similar to that of wild-type FOXM1 (Supplementary Fig. S4B). Taken together, these results demonstrated that ABL1-mediated FOXM1 Y575 phosphorylation is essential for FOXM1 stabilization.

### Ubiquitin-proteasomal degradation of FOXM1 is inhibited by Y575 phosphorylation

**Fig. 5:**
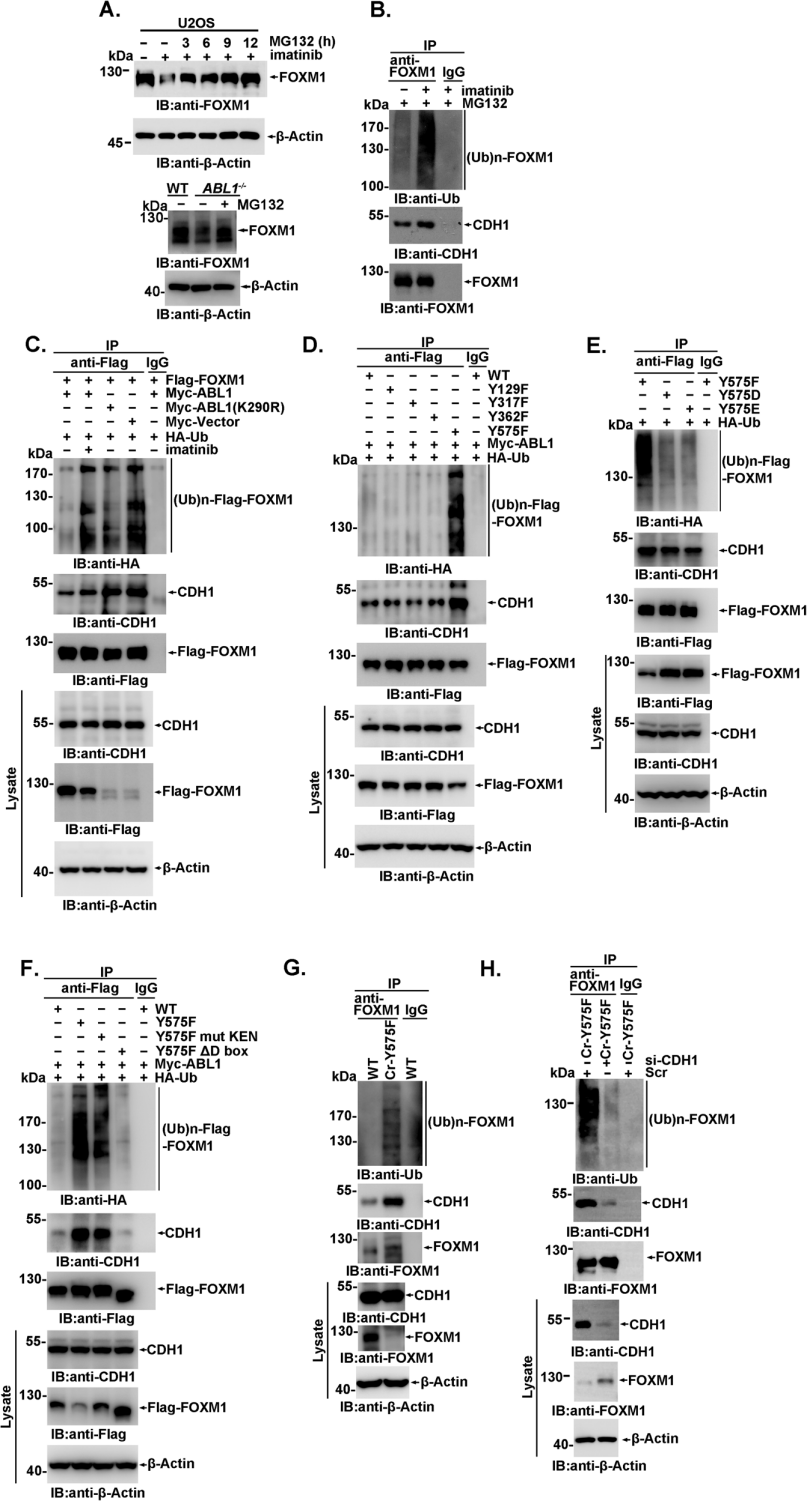
ABL1-mediated Y575 phosphorylation inhibits FOXM1 ubiquitination. **A** Lysates from U2OS cells subjected to MG132 (6 µM) treatment with the indicated time were analyzed by immunoblotting (upper panel). Wild-type and *abl1*^*−/−*^ HeLa cells with the indicated treatment were analyzed by immunoblotting (lower panel). **B** Lysates from U2OS cells subjected to the indicated treatment were analyzed by immunoprecipitation and immunoblotting. 293FT cells transfected with the indicated plasmids (**C**–**F**) or Cr-Y575F and wild-type cells (**G**) were analyzed by immunoprecipitation and immunoblotting. **H** Lysates from Cr-Y575F cells transfected with CDH1 siRNA or scramble siRNA (as a control) were subjected to anti-FOXM1 or IgG (as a control) immunoprecipitation and immunoblotting analysis. In Fig. 5, all IP samples were balanced with the FOXM1 immunoprecipitation level by adjusting the loading volume, while the lysate was balanced with the β-Actin level.

FOXM1 is degraded via the ubiquitin‒proteasome pathway by interaction with CDH1, a coactivator of the E3 ubiquitin ligase anaphase-promoting complex/cyclosome (APC/C) [15, 45], and FOXM1 degradation may be inhibited by Abl-mediated Y575 phosphorylation. Accordantly, downregulation of FOXM1 mediated by imatinib treatment, ABL1 or ABL1/ABL2 depletion, and the mutation Cr-Y575F could be substantially rescued by treatment with the proteasome inhibitor MG132 in U2OS cells, HeLa cells, MCF-7 cells, or MEFs (Fig. 5A and Supplementary Fig. S5A–D). The polyubiquitination of FOXM1 was substantially enhanced by imatinib treatment in the presence of MG132, suggesting that ABL1-mediated phosphorylation prevents FOXM1 degradation via the proteasome pathway (Fig. 5B). Both the ubiquitination of FOXM1 and its association with CDH1 were significantly promoted by ABL1 inhibition or kinase-dead ABL1 mutant expression (Fig. 5B, C), and MG132 could also rescue the downregulation of FOXM1 levels induced by coexpression with Myc-ABL1(K290R) (Supplementary Fig. S5E). Among mutants at four FOXM1 tyrosine phosphorylation sites, only the Y575F mutant demonstrated greatly increased ubiquitination and association with CDH1 (Fig. 5D). Meanwhile, in contrast to FOXM1(Y575F), both of the phosphomimetic mutants FOXM1(Y575D) and FOXM1(Y575E) demonstrated substantially decreased ubiquitination and restored expression levels (Fig. 5E).

It has been reported previously that two consecutive D boxes (aa 1–25) and one KEN box (aa 203–216) are critical for the degradation of FOXM1 by APC/C^CDH1^ [15]. To further address phospho-Y575-regulated FOXM1 degradation, a FOXM1(Y575F) mutant containing either a deleted D box or site-directed mutagenesis of the KEN-box was constructed. A comparison of ubiquitination levels showed that the D-box was the major degron motif responsible for the recognition of FOXM1(Y575F) by CDH1 and its subsequent ubiquitination. The KEN box similarly contributed to this process, albeit to a much lesser extent (Fig. 5F). Both deletion of the D boxes and mutation of the KEN box significantly upregulated FOXM1Y575 expression (Fig. 5F).

In agreement with the significantly decreased stability of FOXM1(Y575F), endogenous FOXM1(Y575F) expressed in the Cr-Y575F cell line showed an enhanced ubiquitination level and increased association with CDH1 (Fig. 5G). To further demonstrate that CDH1 is involved in FOXM1(Y575F) degradation in the Cr-Y575F cell line, endogenous CDH1 expression was knocked down by RNA interference, which resulted in significantly decreased FOXM1 ubiquitination and increased FOXM1(Y575F) levels (Fig. 5H). Notably, the association of CDH1 with APC3, which recruits substrate-binding CDH1 and serves as the center for APC/C regulation, was not significantly altered by ABL1/ABL2 depletion. This result suggests that ABL1 does not regulate the E3 ligase complex directly (Supplementary Fig. S5F). These data collectively showed that ABL1 regulates the ubiquitin-proteasomal degradation of FOXM1 through APC/C^CDH1^ by FOXM1 Y575 phosphorylation.

### The stabilization of FOXM1 by Y575 phosphorylation is crucial for mitotic progression

**Fig. 6:**
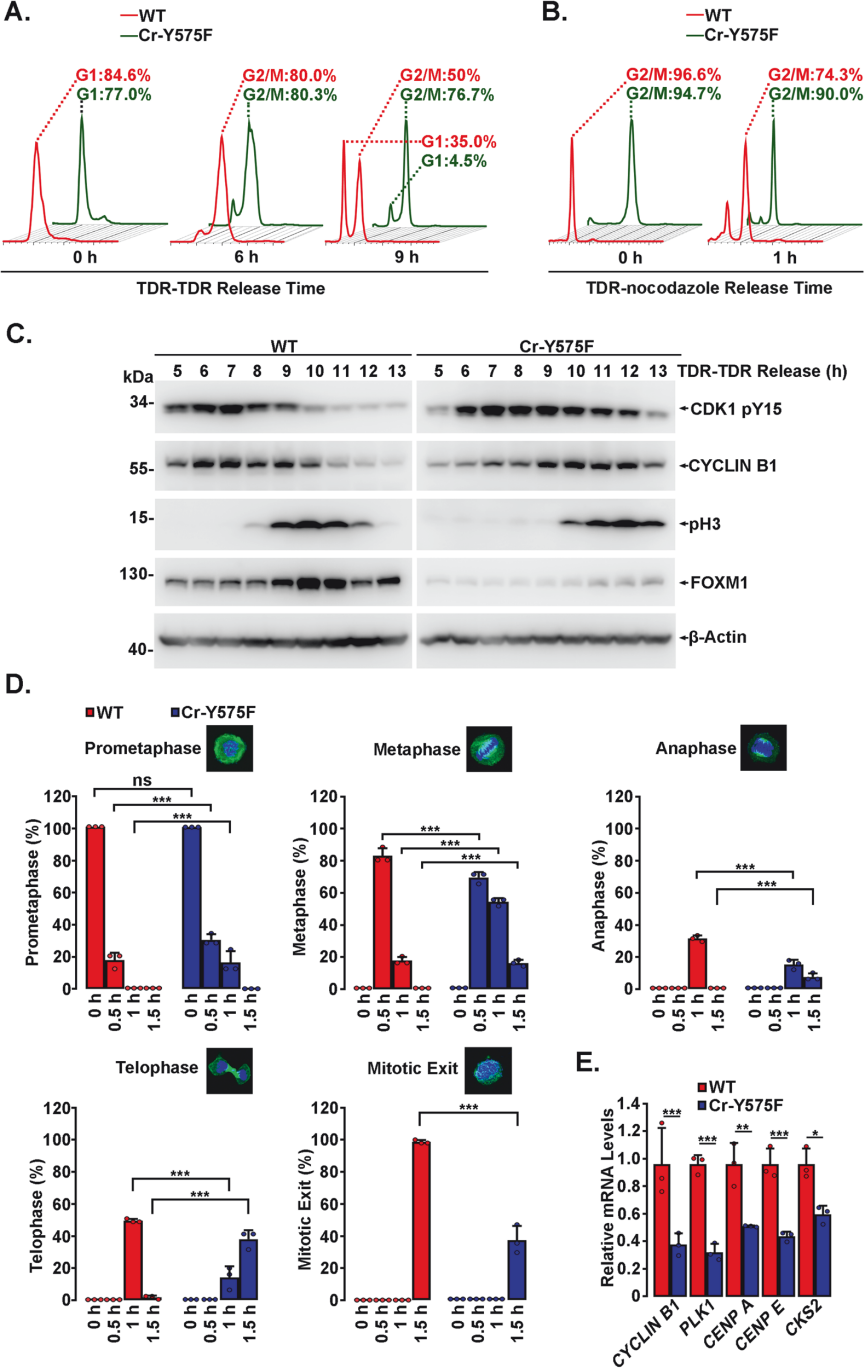
FOXM1 stabilization by Y575 phosphorylation is critical for mitosis. Cr-Y575F and wild-type cells were harvested at the indicated time points after double thymidine release (**A** and **C**) or thymidine-nocodazole release (**B** and **D**), and the cell cycle was analyzed by flow cytometry (**A**, **B**) or immunoblotting with the indicated antibodies (**C**). The mitotic progression of thymidine-nocodazole-synchronized cells was imaged by immunostaining and confocal microscopy. At least 50 cells were counted at each time point, and the percentages of each phase were analyzed and plotted. Representative images of each mitotic phase are also shown (**D**). Data are presented as the mean ± SD of three independent experiments. ns not significant; ***p < 0.001, ANOVA. **E** Cr-Y575F and wild-type cells were harvested after double thymidine release for 6 h, and qRT‒PCR was performed to determine the mRNA levels of the indicated genes. Data shown represent the means ± SD of biological triplicates. *p < 0.05, **p < 0.01, ***p < 0.001, Student’s *t* test.

FOXM1 tyrosine phosphorylation mainly occurred during early G2/M phase (Fig. 3G and Supplementary Fig. S3M), which is coincident with the crucial role of FOXM1. To evaluate the role of FOXM1 Y575 phosphorylation in cell cycle progression, Cr-Y575F and wild-type cells were arrested at the G1/S boundary by double thymidine blockade and then released into fresh DMEM. Cell cycle progression was analyzed by flow cytometry at the indicated time points. Compared with wild-type cells, the Cr-Y575F cell line displayed a significantly prolonged (by approximately 2–3 h) G2/M transition phase (Fig. 6A and Supplementary Fig. S6A), as indicated by the phospho-Y15 level of CDK1 (Fig. 6C). Moreover, compared with wild-type HeLa cells, the Cr-Y575F cell line showed obvious compromise of the accumulation of FOXM1 in G2/M phase, and this change may have been regulated by the cell cycle-dependent phosphorylation of residues other than Y575. Furthermore, the two cell lines were synchronized in prometaphase by a thymidine-nocodazole block and then released. Delayed mitosis progression was also detected in the Cr-Y575F cell line compared with wild-type cells (Fig. 6B and Supplementary Fig. S6B), which was reflected by the spindle morphology in different phases of mitosis (Fig. 6D and Supplementary Fig. S6C). During mitosis, the mRNA levels of many FOXM1-regulated essential mitotic genes, such as *CYCLIN B1, PLK1, CKS2, CENP A* and *CENP E*, were found by qRT‒PCR to be similarly downregulated in Cr-Y575F cells compared to wild-type cells (Fig. 6E). The phosphorylation of FOXM1 by ABL1 had no significant effect on G1/S phase progression (Supplementary Fig. S6D), although FOXM1 has been reported to play a role in G1/S transition. These findings indicated that Y575 phosphorylation is indispensable for not only FOXM1 stability but also FOXM1-dependent mitotic progression.

### ABL1 kinase-regulated FOXM1 stabilization contributes to FOXM1-related tumor development

**Fig. 7:**
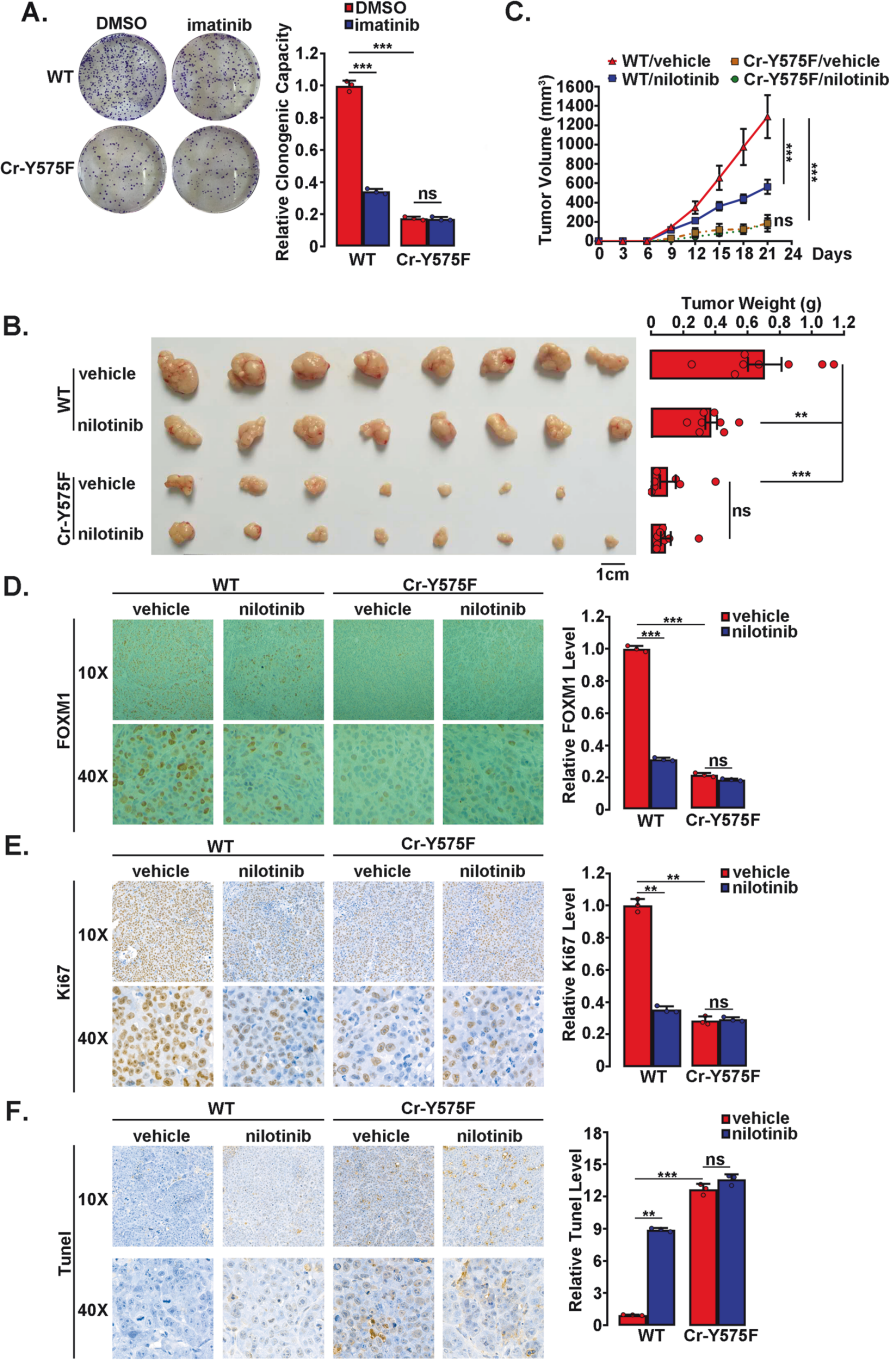
ABL1 kinase-dependent FOXM1 stabilization contributes to tumorigenicity. **A** Colonogenic assay of Cr-Y575F and wild-type cells treated with imatinib (2 µM) or DMSO (as a control). The colony number was counted, and the relative clonogenicity was calculated. The data shown represent the mean ± SD of three independent experiments. ns not significant; ***p < 0.001, ANOVA. **B**, **F** BALB/c null mice subcutaneously injected with Cr-Y575F and wild-type cells were treated with or without the ABL1 inhibitor nilotinib (70 mg/kg) each day. The growth of the xenograft tumors derived from the injected cells was monitored (**C**). After 21 days, the tumor was dissected, imaged (**B**), and weighed (**B**). Data are presented as the means ± SEM for eight mice per group. ns, not significant; **p < 0.01, ***p < 0.001, two-way ANOVA. Representative FOXM1, Ki67 and TUNEL immunohistochemistry of tumors were shown, and relative protein levels were quantified by ImageJ software and plotted in the right panel (**D**–**F**). The data shown represent the mean ± SD of three independent experiments. ns not significant; **p < 0.01, ***p < 0.001, ANOVA.

Since high FOXM1 expression plays a key role in tumorigenesis, we then sought to determine the effect of Y575 phosphorylation on FOXM1 tumorigenesis capacity. In the colony growth assay, we found that Cr-Y575F cells demonstrated a significantly attenuated colony formation capacity. An approximately 80% decrease in colony formation was observed in Cr-Y575F vs. Wild-type cells. Accordingly, imatinib-induced ABL1 inhibition also resulted in a 60% decrease in colony formation in wild-type cells but not in Cr-Y575F cells (Fig. 7A). Next, we established a xenograft tumor model by subcutaneously injecting Cr-Y575F or wild-type cells into the anterior limbs of nude mice. Simultaneously, mice were intragastrically administered nilotinib or solvent only as a control each day, and mouse weight and tumor size were monitored. In agreement with the colony formation results, inhibited growth and decreased size of xenograft tumors developed from Cr-Y575F cells were observed (Fig. 7B, C). Nilotinib treatment markedly inhibited tumor growth in mice inoculated with wild-type cells but had no effect on Cr-Y575F cell-inoculated mice. Furthermore, the immunohistochemical analysis of xenograft tumors also demonstrated impaired FOXM1 expression in Cr-Y575F cell line-derived tumors, as well as in wild-type cell-derived tumors subjected to nilotinib administration (Fig. 7D), indicating a strong correlation between ABL1-regulated FOXM1 expression and tumor development. To further evaluate the level of tumor cell proliferation and apoptosis, tumor slices were subjected to Ki67 and TUNEL staining. Compared with the vehicle control, a significantly decreased Ki67 level (Fig. 7E) and enhanced TUNEL signal (Fig. 7F) were observed in nilotinib-treated or Cr-Y575F cell-derived tumors.

To further address the role of ABL1-FOXM1 pathway in breast cancer, we next extended our observations to MCF-7 cells. A FOXM1 Cr-Y575F MCF-7 cell line was established, and the cells exhibited decreased FOXM1 phosphorylation and lower FOXM1 expression levels (Supplementary Fig. S7A), an enhanced FOXM1 ubiquitination level and increased FOXM1:CDH1 association (Supplementary Fig.S7B) compared to the wild-type MCF-7 cells. Moreover, compared to wild-type cells, FOXM1 Cr-Y575F MCF-7 cells exhibited significantly slower tumor growth (Supplementary Fig. S7C, D). Consistently, doxorubicin-induced apoptosis was greater in FOXM1 Cr-Y575F cells than in the wild-type cells, as shown by increased cleavage of CASP3 and PARP-1 (Supplementary Fig. S7E). We also investigated the ABL1-FOXM1 pathway in other cancer cell lines. A total of nine cancer cell lines were subjected to asciminib treatment, and remarkably impaired stability of FOXM1 and decreased expression were observed in most of these cancer cell lines including prostatic cancer LNCaP, lung cancer A549, hepatocellular carcinoma HuH7, pancreatic cancer MIA PaCa-2, and breast cancer BT-20 and SK-BR-3, whereas asciminib treatment could not significant impact FOXM1 expression in neuroblastoma SH-SY5Y, ovarian cancer SK-OV-3 and breast cancer MDA-MB-231 cell lines (Supplementary Fig. S8). We speculated that the tumorigenesis of tumor cells with unaffected FOXM1 expression by asciminib may not be much altered by c-ABL inhibitor treatment too.

**Fig. 8:**
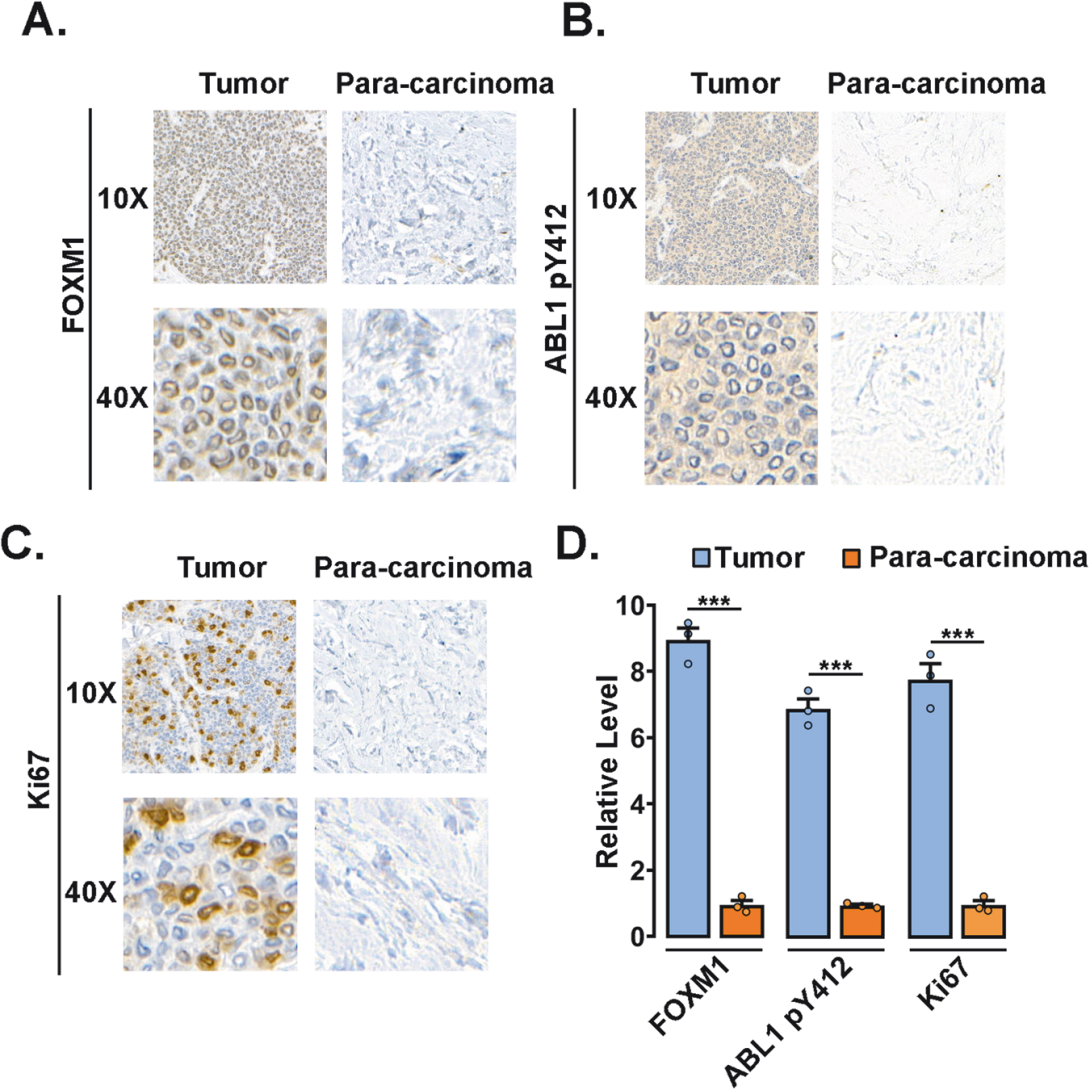
The activation of the ABL1-FOXM1 pathway in breast cancer. **A**–**D** Representative FOXM1, ABL1p-Y412 and Ki67 immunohistochemistry of tumors or para-carcinoma were shown, and relative protein levels were quantified by ImageJ software and plotted. The data shown represent the mean ± SD of three independent experiments. ***p < 0.001, ANOVA.

The activation of the ABL1-FOXM1 pathway in breast cancer was further investigated with patient samples. The FOXM1 expression, the level of ABL1 phosphorylated at Y412 (pY412), and Ki67 level were significantly increased in tumor tissues compared to paracarcinoma tissues (Fig. 8A–D), indicating an essential role of ABL1 activation in FOXM1 expression and cell proliferation. Taken together, these findings in tumor cells suggest that ABL1-mediated FOXM1 phosphorylation and stabilization contribute to tumor development by promoting cell proliferation and suppressing tumor cell apoptosis.

## Discussion

High expression or activation of ABL1 or FOXM1 overexpression are consistently observed in various cancers [4, 24, 26, 46–48], but the possible links between them are largely unknown. In this study, we revealed that ABL1 kinase regulates FOXM1 phosphorylation and maintains FOXM1 abundance by preventing its degradation via the ubiquitin-proteasomal pathway. A significant decrease in FOXM1 levels was observed by ABL1 kinase inhibition and gene knockout or knockdown, indicating that the kinase activity of ABL1 is indispensable for high FOXM1 expression. Posttranslational modifications of FOXM1, such as serine/threonine phosphorylation [2, 12], ubiquitination and deubiquitination [9, 10], sumoylation [49, 50] and acetylation [51], play important roles in FOXM1 activation and expression. However, the tyrosine phosphorylation of FOXM1 was rarely reported until now.

Four pTyr sites in FOXM1 were identified in this study from cells coexpressing FOXM1 and ABL1, while the previously reported FOXM1 pTyr site, pY517, was identified from cells that arrested in mitosis and were subjected to a fold change selection of cell cycle kinase-specific inhibitors [52]. FOXM1 tyrosine phosphorylation has also been reported in K562 cells (unpublished data), a BCR-ABL-positive human chronic myeloid leukemia cell line. Unlike ABL1, the fusion BCR-ABL kinase is constitutively activated and exclusively located in the cytoplasm, which renders its function very different from that of ABL1. Additionally, after trypsin digestion, Y575 was found to be located within a relative long peptide (29 aa, R.WAAELPFPADSSDPASQLSY_575_SQEVGGPFK). However, the accuracy of resolving long peptides with different MS/MS systems might differ. This may explain why the pTyr residues that we identified have not been reported elsewhere. To evaluate the possibility of an artificial result, a FOXM1 pY575-specific polyclonal antibody was generated using the Cys-PASQLSY(p)SQEVGG peptide as an immunizing antigen, and the tyrosine phosphorylation of FOXM1 was further confirmed with a FOXM1-pY575 site-specific antibody in vivo (Fig. 3F, G and Supplementary Fig. S3L) and in vitro (Supplementary Fig. S3K).

A previous study reported that PLK1-mediated FOXM1 S715/S724 phosphorylation activates FOXM1 and shields it from proteolytic degradation [2]. Recently, Xu et al. reported that PLK1 is phosphorylated and activated by ABL1 directly during G2/M phase [53], suggesting that ABL1 is involved in FOXM1 phosphorylation not only by direct phosphorylation but also by potentiating PLK1-mediated serine phosphorylation. Thus, the synergetic modification of FOXM1 by ABL1 and PLK1 may contribute to the full activation and stabilization of FOXM1, facilitating its mitotic function. Notably, FOXM1 is also phosphorylated and stabilized by CDK4/6 in a similar way, thereby enhancing the expression of G1/S phase genes and promoting S phase entry [12]. At the late S and G2 phases, when CDK4/6 is inactive, the mechanism shifts to ABL1 together with other kinases, such as PLK1, to maintain FOXM1 abundance; thus, the variation in FOXM1 expression is achieved via different regulators activated at different stages of the cell cycle.

The ubiquitination of FOXM1 is mediated by several E3 ligases, including FBW7 [54], SCF-FBXO31 [13], Cul4-VprBP [55], RNF168 [56] and APC/C^CDH1^ [15]. Here, APC/C^CDH1^ was identified to be involved in the ubiquitination of FOXM1(Y575F) by the enhanced association of the CDH1 adapter with FOXM1(Y575F). ABL1 is activated in S phase and subjected to CDK1-mediated hyperphosphorylation in M phase. CDK1 activity declines through anaphase and telophase, as does the hyperphosphorylation and activation of ABL1 kinase [57]. It has been reported that the TAD of FOXM1 can be suppressed by direct interaction with the NRD of FOXM1 [58]. Cyclin A/E-CDK2-mediated FOXM1 phosphorylation at T600, T611 and S638 relieves repression of the TAD by the NRD and restores TAD transactivation activity during G2 phase [59], while ABL1 phosphorylates FOXM1 pY575 near the TAD in early G2/M phase, which may also be involved in this process. We proposed that dissociation of the NRD and TAD results in a conformational change that may interfere with the degradation function of N-terminal degrons.

Therefore, at the end of M phase, both activation of the APC/C complex and elimination of ABL1-mediated phosphorylation (Fig. 3G) contribute to the rapid degradation of FOXM1, which is critical for anaphase progression as it shuts down the transcriptional activation of mitotic regulators during the end stage of mitosis [60]. Compared with the wild-type cell line, Cr-Y575F cells demonstrated significantly delayed mitotic progression, which was caused by compromised FOXM1 accumulation and the subsequent insufficient supply of mitotic regulators, such as CYCLIN B1, PLK1, CENP A and CENP F. Moreover, a significantly reduced FOXM1 level in Cr-Y575F cells was observed throughout the whole cell cycle and was not limited to the mitotic phase. We presumed that CDH1-mediated APC/C ligase activation beyond mitosis may be responsible for this constitutive degradation of FOXM1(Y575F), since APC/C^CDH1^ ligase is not fully inactivated after cell exit mitosis but still maintains moderate and fluctuating activity during the nonmitotic phase [61–63]. It is also of interest to clarify the possible contribution of c-Abl:FOXM1 to FOXM1 conformation change and activation. we tried but failed since no FOXM1 structure was available, and the confidence of AlphaFold predicted structure is too low to be used for such analysis.

To clarify the subcellular region that ABL1-mediated phosphorylation of FOXM1 occurred, we investigated the FOXM1 phosphorylation in cytoplasm and nuclear in HeLa and MCF-7 cells, and the data revealed that phosphorylated FOXM1 accumulated majorly in the nucleus (Fig. 3B and Supplementary Fig S3C). Considered that ABL1 interacted with FOXM1 both in the cytoplasm and nucleus, the phosphorylation may occur in the nucleus, and possibly in the cytoplasm before entering the nucleus like many other transcription factors.

ABL1 regularly maintains an autoinhibited configuration and lower kinase activity, which could be activated by stress stimuli such as oxidative stress, irradiation induced DNA damage, and substrate interactions [64, 65]. As a cell cycle-dependent kinase, ABL1 is activated in S phase and the increase of ABL1 kinase activity was only observed for the nuclear ABL1 pool [66]. The cytoplasmic ABL1 kinase activity did not change throughout the cell cycle [67]. ABL1 was subjected to CDK1-mediated hyperphosphorylation in G2/M phase [57], which may alter its property in vivo to phosphorylate specific substrates, although no effect on the measurable kinase activity in vitro was observed [57], ABL1 substrates that binds to SH2 and SH3 are often to be its allosteric activators [68]. For example, the c-Jun transcription factor activates nuclear ABL1 by binding with ABL1, in turn is phosphorylated by ABL1 [69]. These observations suggested that ABL1 mediates the cell cycle dependent FOXM1 phosphorylation by FOXM1:ABL1 interaction, in the absence of DNA damage.

Generally, activation of nuclear ABL1 lead to apoptosis in response ionizing radiation induced DNA damage, which was dependent on the activation of the ataxia telangiectasia mutated protein (ATM), DNA-PK, and the phosphorylation of P53 and P73 [65]. Activation of ABL1 in responses to the activation of receptor tyrosine kinases (RTKs), chemokine receptors, or the inactivation of inhibitory proteins has oncogenic effect [34]. In this study, FOXM1 may be phosphorylated by cytoplasmic ABL1 in response to stimuli and induces tumorigenesis, while FOXM1 phosphorylated in the nucleus contributed to cell proliferation, particularly in the cells with ABL1 overexpression and activation, especially in the tumor cells.

Because of its high expression and crucial roles in tumorigenesis, FOXM1 has been determined to be a cancer therapeutic target. Inhibition of FOXM1 expression seems to be a reasonable approach to antagonize tumors with FOXM1 overexpression, and most strategies inhibit FOXM1 by repressing its promoter directly. Here, we showed that a ABL1 kinase inhibitor extensively used in CML therapy suppressed solid tumor growth. This finding partially explains the mechanism underlying the protumorigenic role of ABL1 kinase in cervical and breast cancer investigated in this study. We also observed that the tumorigenic capability of Cr-Y575F cells was significantly lower than that of wild-type cells treated with the ABL inhibitor, indicating that ABL1 kinase may not be the only tyrosine kinase responsible for Y575 phosphorylation. As shown in Supplementary Fig. S3A, imatinib treatment could not completely eliminate the tyrosine phosphorylation of FOXM1, which may support this hypothesis. Collectively, our findings suggest that the inhibition of FOXM1 Y575 phosphorylation leads to significant suppression of tumor growth, suggesting that ABL1 kinase inhibitors could be potential clinical agents for FOXM1-overexpressing tumor therapy.

## Methods

### Antibodies

HRP-anti-Flag (Sigma, Cat# A8592), HRP-anti-Myc (Santa Cruz, Cat# SC-40), anti-β-actin (Santa Cruz, Cat# SC-1616), HRP-anti-HA (Sigma, Cat# H9658), HRP-anti-p-Tyr (Millipore, Cat# 16-105), HRP-anti-GST (Proteintech, Cat# HRP-66001), HRP-anti-Ub(Santa Cruz, Cat# sc-8017), anti-α-Tubulin (Sigma, Cat# T9822), anti-FOXM1 (Santa Cruz, Cat# SC-502), anti-FOXM1 (Santa Cruz, Cat# SC-376471), anti-ABL1 (Santa Cruz, Cat# SC-131), anti-pH3 (CST, Cat# 9701S), anti-CYCLINB1 (Novus, Cat# 2061A), anti-CDH1 (Novus, Cat# DCS-266), anti-Cleaved CASP3 (CST, Cat# 9664S), anti-Cleaved PARP-1 (Santa Cruz, Cat# SC-56196), anti-APC3 (proteintech, 10918-1-AP), anti-Ki67 (CST, Cat# 9129), anti-ABL1 pY412 (phosphosolutions, AP1271), anti-rabbit IgG secondary antibodies (GE, Cat# NA934V), anti-mouse IgG secondary antibodies (GE, Cat# NA931V), goat anti-mouse IgG LCS (Abbkine,Cat# A25012), and FITC-conjugated goat anti-mouse IgG secondary antibodies (ZSGB-BIO, Cat# ZF-0312).

### Chemicals and reagents

Flag M2-Agarose Affinity Gel (Sigma, A2220), Myc M2-Agarose Affinity Gel (Sigma, E6654), mouse IgG-Agarose beads (Sigma, A0919), protein A Sepharose (GE, 17-0780-01), DMEM (Sigma, RNBJ5741), fetal bovine serum (Tecono, F801-500), MG132 (Sigma, M8699), CHX (MedChemExpress, HY-123320), doxorubicin (Sigma, D1515), imatinib (Novartis, 220127-57-1), nilotinib (Novartis, S0045), asciminib (Selleck, S8555), Cas9 protein (Invitrogen, A36499), Lipofectamine 3000 (Invitrogen, L3000-015), leupeptin(Roche, 04693132001), chemiluminescence agent (Millipore, WBKLS0500), SYBR Green qPCR Super Mix (BIO-RAD, 172-5121), thymidine (Sigma, T9250), nocodazole (Sigma,M1404-10MG), propidium iodide (Sigma, P4170), the TransIT-X2 Dynamic Delivery System (Mirus,MIR6000), DMSO (Sigma, D2650), Triton X-100 (Sigma, T8787-50ML), DAPI(Sigma, D8417), and the following critical commercial assays: the QuikChange site-directed mutagenesis kit(SBS Genetech Co., Ltd., SDM-15), an RNA isolation kit (QIAGEN, 74104), a reverse transcription kit(Promega,A5001), and the Duo-Link kit (Sigma, Duo92008), NE-PER^TM^ Nuclear and Cytoplasmic Extraction Reagents (Thermo Scientific, 78833).

### Cell culture and transfection

WT, *ABL1* knockout and Cr-Y575F HeLa cells, MCF-7, U2OS cells, 293FT cells, A549 cells, HuH7 cells, MIA PaCa-2 cells, WT and *ABL1/ ABL2* knockout MEFs were cultured in DMEM medium, SK-OV-3 cells and SK-BR-3 cells were cultured in McCoy’s 5A medium, BT-20 cells and SH-SY5Y cells were cultured in MEM medium, LNCaP cells and MDA-MB-231 cells were cultured in RPMI-1640 medium, containing 10% fetal bovine serum, 2 mM L-glutamine, 100 IU/ml penicillin and 100 µg/ml streptomycin. The cells were verified and were free of mycoplasma contamination based on the results of the Mycoplasma Stain Assay Kit (Beyotime). The cells were treated with imatinib, MG132 or CHX as indicated in the text. Transient transfection was performed with Lipofectamine 3000 according to the manufacturer’s instructions.

### Immunoprecipitation and immunoblotting

Cells were harvested and lysed in lysis buffer (150 mM NaCl, 0.5 mM EDTA, 50 mM Tris-HCl (pH 8.0), 1% Nonidet P-40, 10 µg/ml leupeptin). Soluble proteins were subjected to immunoprecipitation with anti-FLAG, anti-Myc or the indicated antibodies and protein A Sepharose for 2 h at 4 °C, and normal rabbit IgG or normal mouse IgG was used as a control. In addition, an aliquot of the total lysate (5%, v/v) was included as a control. Immunoblotting was performed by using the indicated antibodies, and the antigen-antibody complexes were visualized by enhanced chemiluminescence (ECL). Data shown are representative of three independent experiments. Original Western blots are provided in the Supplementary Materials.

### LC‒MS/MS analysis

Flag-FOXM1 and Myc-ABL1 plasmids were coexpressed in 293FT cells, Flag-tagged FOXM1 immunoprecipitates were prepared from whole cell lysates and resolved by SDS‒PAGE, and the protein bands were excised. After trypsinization, phosphopeptides were enriched with TiO2 resin. LC-electrospray ionization-MS/MS-resolved peptides were analyzed using a Q-TOF2 system, and the data were compared against SWISSPROT using the Mascot search engine for phosphorylation.

### GST pulldown assay

GST-ABL1-SH2, GST-ABL1-SH3 or GST bound to glutathione-Sepharose resin control was incubated with cell lysates containing Flag-tagged FOXM1 for 2 h at 4 °C, and the resins were washed 3 times with lysis buffer. The resin-bound complexes were boiled, separated by SDS‒PAGE, and then analyzed by immunoblotting or Coomassie blue staining. Data shown are representative of three independent experiments.

### Plasmid construction

Complementary DNA encoding human FOXM1 and ABL1 (the 1b isoform) was generated by PCR amplification and cloned into the pcDNA3-base Flag vector or pCMV-Myc-vector, respectively. Flag-FOXM1(Y129F), Flag-FOXM1(Y317F), Flag-FOXM1(Y362F), Flag-FOXM1(Y575F), and Myc-ABL1(K290R) mutant plasmids were generated by using a Quick-change Site-directed Mutagenesis Kit according to the manufacturer’s instructions.

### Quantitative RT‒PCR

Total RNA was extracted using an RNA isolation kit, and cDNA was generated by reverse transcription. Quantitative real-time PCR was performed using SYBR Green qPCR Super Mix. GAPDH or β-actin was used as a reference gene. Data shown are representative of three independent experiments. The primers used are listed in Supplementary Tables S1 and S2.

### Generation of *ABL1* gene knockout and Cr-Y575F cell lines

The sgRNA was designed using the online CRISPR Design Tool (http://tools.genome-engineering.org) and cloned into the pSpCas9 (BB)-2A-Puro vector for coexpression with Cas9. Then, the plasmids were transfected into HeLa cells. For Cr-Y575F cell line generation, the plasmid was cotransfected with a single-stranded oligo donor to generate a precise point mutation in FOXM1. Seventy-two hours after transfection, the cells were selected with puromycin until colonies were generated. DNA was extracted from clonal lines by PCR amplification, and then the correct clonal lines were selected by sequencing analysis. The genomic mutation in selected cell lines was further verified by T-vector sequencing. At least 30 T-vector clones were sequenced, all of which showed correct nucleotide acid substitution (a clear sequencing peak with only little baseline noise at the target site), indicating that all genomic copies had been edited successfully. The sgRNA sequence and the template oligo sequence are listed in Supplementary Tables S3 and S4.

The control wild-type cells were generated in a similar way by pSpCas9(BB)-2A-Puro stable transfection.

For Cr-Y575F MCF-7 cell line generation, the sgRNA, template oligo and Cas9 protein were electro-transfected into cells. After cultured for 24 h, the cells were dispersed into single-cell suspension by trypsinization and cultured in 96-well plates by limited dilution, the correct clonal lines were selected by sequencing analysis.

### FOXM1-pY575-specific polyclonal antibody generation

New Zealand rabbits were subcutaneously injected at multiple sites with the Cys-PASQLSY(p)SQEVGG peptide as an immunizing antigen. After booster immunization every two weeks for a total of four times, the FOXM1-Y575-specific phosphorylated/nonphosphorylated antibodies were purified from rabbit serum by peptide affinity chromatography. The reactivity and specificity of the antibody were verified by ELISA (Supplementary Fig. S3J), generation of the Y575F mutation (Supplementary Fig. S3K), and antibody blocking (Supplementary Fig. S3L).

### Cell synchronization and flow cytometry assay

For the double thymidine block, the cells were arrested with thymidine (2 mM) for 12 h. The medium was removed, and the cells were washed 3 times with PBS buffer, released into fresh medium for 10 h and arrested with thymidine for another 12 h. For prometaphase arrest, the cells were treated with thymidine (2 mM) for 12 h, removed for 10 h and arrested with nocodazole for 12 h, then shaken off, mixed with fresh medium and collected at the indicated time.

The collected cells were washed with PBS once, resuspended in PBS containing 5% FBS, and then fixed in PBS containing 5 ml 70% ethanol at −20 °C overnight. The cells were stained with 5 µg/ml propidium iodide (PI) for 30 min after treatment with 20 µg/ml RNase and then subjected to flow cytometry analysis. ModFitLT and FlowJo software were used to evaluate the fluorescence intensity. Data shown are representative of three independent experiments.

### In vivo tumorigenicity assay and immunohistochemistry

For the animal experiments, female BALB/c null mice were purchased from Beijing Vital River Laboratory Animal Technology Co., Ltd. and were randomly divided into groups and injected subcutaneously in the right flanks with 2 × 106 cells. Eight mice were used in each group and subjected to nilotinib (70 mg/kg), asciminib (30 mg/kg) or vehicle treatment via intragastric administration. The nilotinib and asciminib were diluted at DMSO with the concentration of 15 μg/μl and 60 μg/μl, then diluted at saline with the final concentration of 5.6 μg/μl and 2.4 μg/μl, respectively. 200 μl of inhibitor diluent was administered per mice each day. Once palpable tumors were established 6 days later, tumor volumes were measured every 3 days, and tumor weights were determined after dissection. The experiments were completed in the experimental animal center of the Academy of Military Medical Sciences, China, and were approved by the Institutional Ethics Committee of Military Medical Science. The experiments were performed in a blinded manner.

In this study, five pairs of tumor and para-carcinoma tissues from five patients (ER+/PR+ x1, ER+/PR− x2, ER−/PR− x2) diagnosed with breast cancer in Chinese PLA General Hospital, Beijing, China, were used for immunohistochemistry analyses, with informed consent and ethics committee approval. The evaluation of the tissue sample was performed in a blinded fashion by two pathologists.

Samples were fixed in 4% paraformaldehyde and embedded in paraffin wax, and the paraffin-embedded tissue sections were deparaffinized by treating with Xylene for 8 min for three times, absolute ethyl alcohol for 8 min for twice, 90%, 80%, 60% ethyl alcohol and distilled water for 8 min. After pretreated with 3% H_2_O_2_ for 20 min, the antibody-binding epitopes of the antigens were retrieved by high pressure and heat treatment for 3 min in the present of EDTA (pH8.0), after washing three times with PBS, the sections were then preincubated with 10% goat serum at room temperature to block nonspecific binding. After washing three times with PBS, the tissues were incubated with primary antibodies overnight at 4 °C, washed three times with PBS, then incubated with HRP-conjugated secondary antibodies for 1 h at room temperature and visualized with DAB. Sections were counterstained with hematoxylin, and the primary antibodies and secondary antibodies were diluted at 1:50 and 1:100, respectively.

The DAB chromogen integrated density value of the immunohistochemically stained tissues, were quantified by ImageJ software. The relative staining level were statistically analyzed and plotted by GraphPad Prism 8 software. The IHC staining of one pair of samples (ER+/PR−) was showed in the manuscript since the other four pairs of samples demonstrated similar IHC staining patten.

### RNAi

The siABL1 target sequence was 5’-GGGAAAUUGCUACCUAUGG-3’, siFOXM1 was 5′-CAACAGGAGUCUAAUCAAG-3, the control siRNA sequence was 5’-UUCUCCGAACGUGUCACG-3’, and transfection was performed with the transit-X2 system according to the manufacturer’s instructions. Seventy-two hours later, the cells were collected for western blotting analyses.

### Clonogenic assay

A total of 2000 wild-type cells or Cr-Y575F cells were seeded in six-well plates and treated with imatinib (2 µM) or DMSO. Ten days later, the cells were fixed with 4% paraformaldehyde for 20 min and stained with crystal violet, and then images were acquired with a camera. Representative results are shown from experiments repeated three times.

### Confocal microscopy

Cells were fixed with 4% paraformaldehyde for 20 min, permeabilized with 0.2% Triton X-100 for 10 min at room temperature, and nonspecifically blocked with PBS buffer containing 1% goat serum for 1 h. The cells were then incubated with primary antibody for 1 h and secondary antibody for another 1 h at room temperature. Nuclei were stained with DAPI.

For the Duo-Link experiment, assays were performed according to the manufacturers’ instructions, and samples incubated with anti-FOXM1 or anti-ABL1 antibody only were used as controls. Images were acquired using a Zeiss LSM 800 confocal microscope. Images were randomly obtained using the DAPI channel to avoid bias in th selection of cells with particular phenotypes before other channels were used for imaging.

### Far western assay

Flag-FOXM1 plasmids were transiently expressed in 293FT cells, and soluble proteins were subjected to immunoprecipitation with anti-FLAG Sepharose beads or normal mouse IgG Sepharose beads (as a control). The beads were washed three times and boiled. Bead-bound proteins were separated by SDS‒PAGE and transferred to PVDF membranes. Then, the membranes were incubated with purified GST-ABL1 SH2, GST-ABL1 SH2 or GST proteins (as a control) at 4 °C overnight and analyzed by immunoblotting with HRP-anti-GST or anti-Flag antibodies. Coomassie blue staining was performed to evaluate the GST-ABL1 SH2, GST-ABL1 SH2 and GST proteins. Representative results are shown from experiments repeated three times.

### Quantification and statistical analysis

All experiments were replicated at least three times, and statistical analysis was carried out using GraphPad Prism 7. Statistical tests were performed using unpaired wo-tailed Student’s *t* test or one-way or two-way ANOVA for differential comparison between two or more groups. No data were excluded from the analyses unless indicated. Data were considered significant when *p* < 0.05.

## Supplementary information

Supplementary Data

original western blots

## Data Availability

All data generated or analyzed are included in the article and its supplementary files, and available from the corresponding author upon request.
