# Supplementary material for: ABL1-mediated phosphorylation promotes FOXM1-related tumorigenicity by Increasing FOXM1 stability
Source: Cell Death Differ. 2024 Jul 26;31(10):1285–301. doi: 10.1038/s41418-024-01339-w (PMC11445503; doi:10.1038/s41418-024-01339-w)
Supplement: Supplementary file 1 — Supplementary Data [file 41418_2024_1339_MOESM1_ESM.pdf]

Supplementary Fig.S1

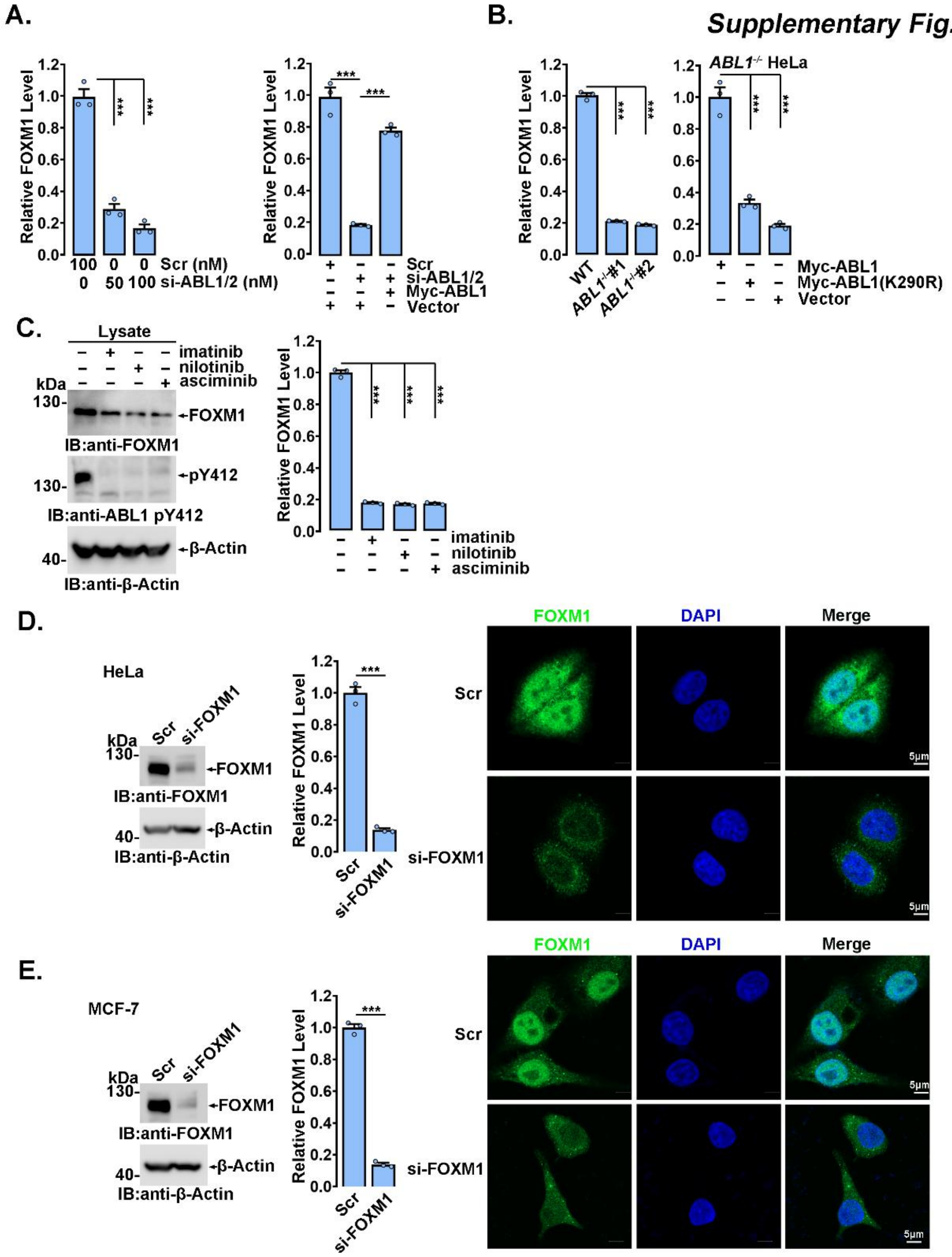

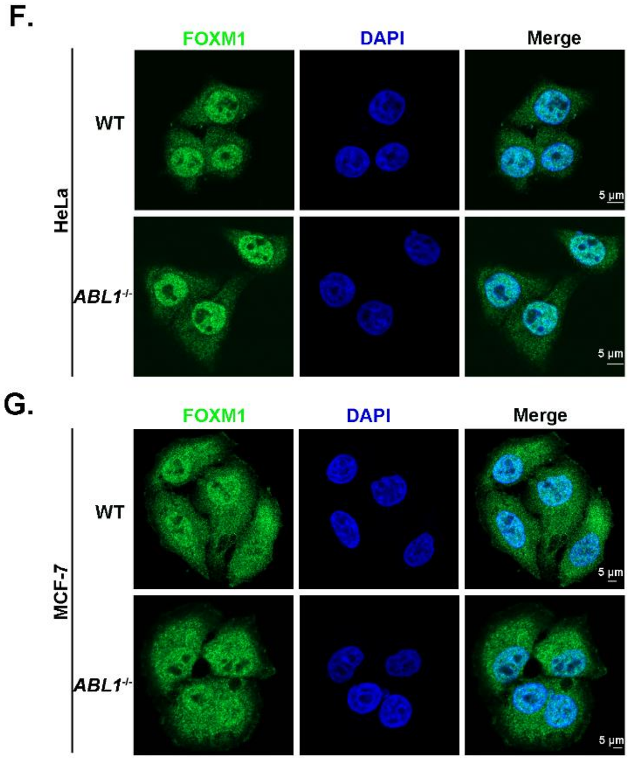

Supplementary Fig.S1. ABL1 regulates FOXM1 expression.

(A and B) The relative protein levels from Fig.1E and F were quantified and statistically analyzed, and represented as mean±S.D. of three independent analysis. ***p<0.001, ANOVA.

(C) HeLa cells were treated with imatinib, nilotinib or asciminib, and subjected to immunoblotting analysis. The relative protein levels were quantified and statistically analyzed, and represented as mean±S.D. of three independent analysis. ***p<0.001, ANOVA.

(D and E) Immunoblotting and immunofluorescent staining analysis of FOXM1 in FOXM1 siRNA or scramble cells with the anti-FOXM1 antibody. Data shown represent the means±SD of biological triplicates. ****p*<0.001, Student's t test.

(F and G) HeLa (F) or MCF-7 (G) cells were incubated with primary anti-FOXM1 antibody then incubated with FITC- linked secondary antibody. Nuclei were stained with DAPI. The Representative images were shown.

Supplementary Fig.S2

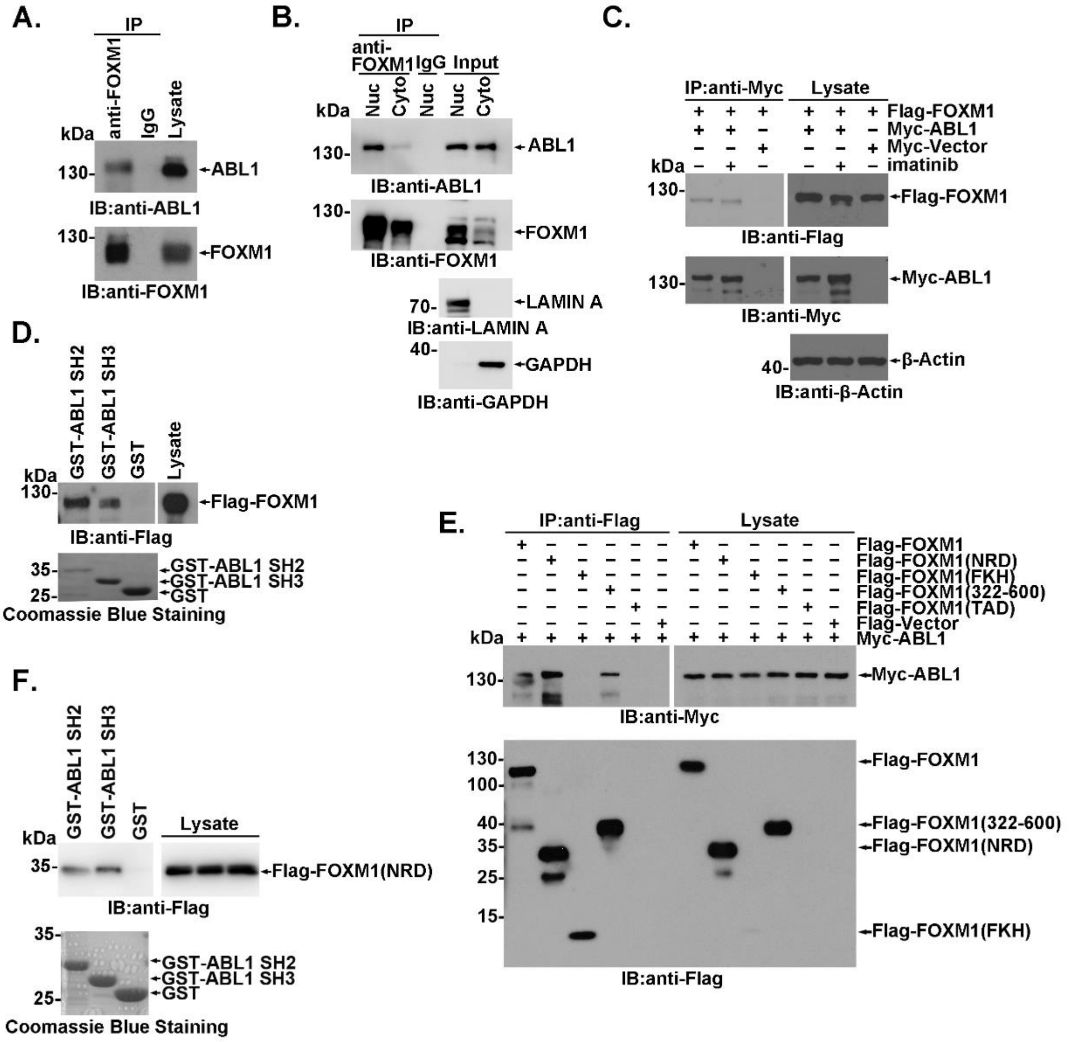

Supplementary Fig.S2 FOXM1 interacts with ABL1.

(A) Total lysates from HeLa cells were subjected to anti-FOXM1 or IgG (as a control) immunoprecipitation, and the immunoprecipitates were analyzed by immunoblotting. (B) Nuclear or cytoplasmic fractions from MCF-7 cells were subjected to anti-FOXM1 or IgG immunoprecipitation, and the immunoprecipitates were analyzed by immunoblotting.

(C) 293FT cells transfected with the indicated plasmids were analyzed by immunoprecipitation and immunoblotting. (D) Lysates of 293FT cells expressing Flag-FOXM1 were incubated with GST-ABL1 SH2, GST-ABL1 SH3 or GST-conjugated glutathione agarose beads, and then the absorbates were analyzed by immunoblotting and Coomassie blue staining. (E) The immunoprecipitation assay between Flag-FOXM1 functional domains and Myc-

ABL1. NRD: N-terminal repressor domain; FKH: Forkhead winged helix domain; TAD: C-terminal transactivation domain. (F) GST pull-down assay in 293FT cells expressing the Flag-FOXM1 NRD domain as described in D.

Supplementary Fig.S3

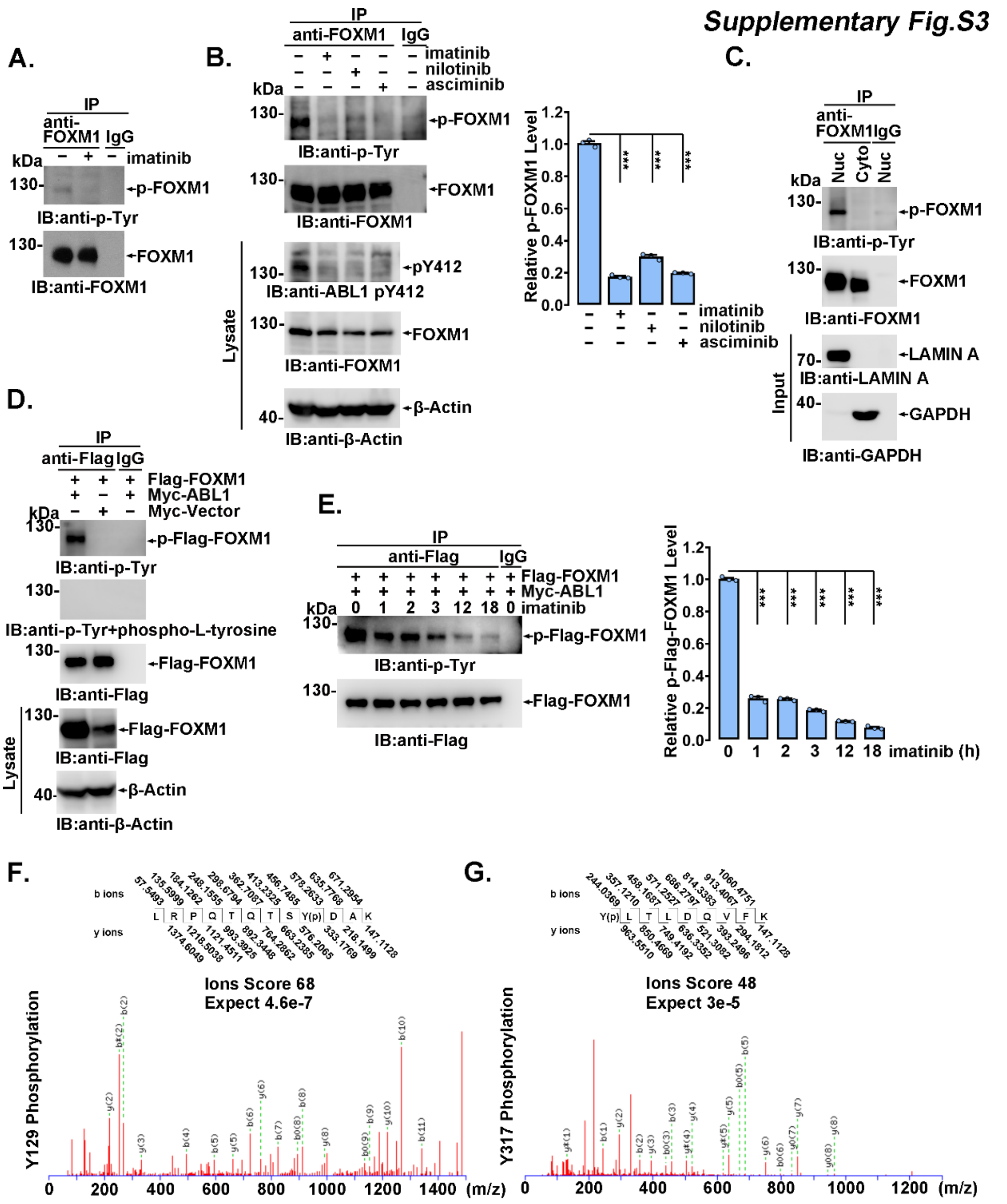

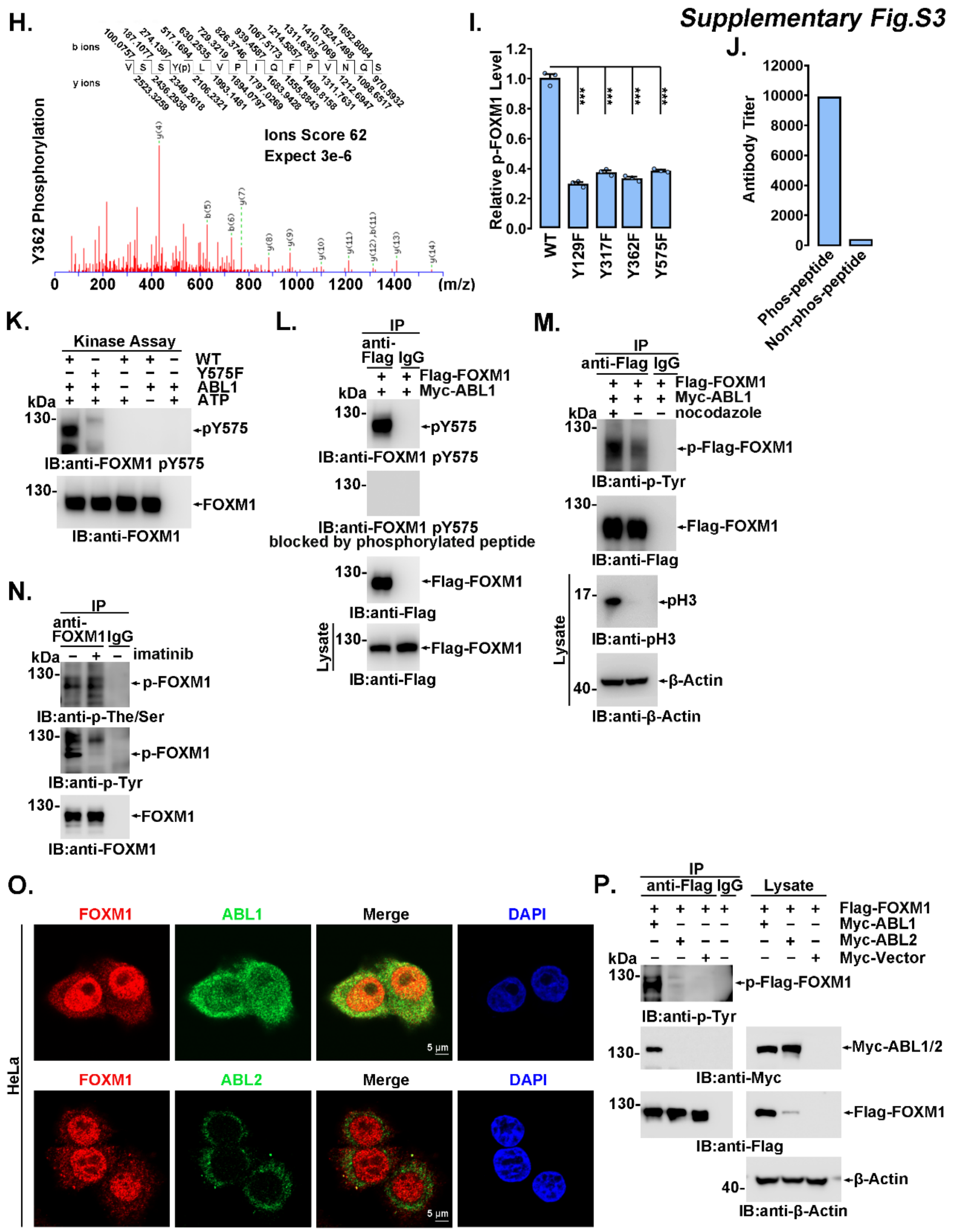

Supplementary Fig.S3 Analysis of tyrosine phosphosites on FOXM1.

(A) U2OS cells treated with or without imatinib were analyzed by immunoblotting. (B) MCF-7 cells were treated with imatinib, nilotinib or asciminib, and subjected to immunoprecipitation and immunoblotting

analysis. The relative p-FOXM1 levels were quantified and statistically analyzed, and represented as mean±SD of three independent analysis. ***$p<0.001$, ANOVA. (C) Nuclear or cytoplasmic fractions from MCF-7 cells were subjected to anti-FOXM1 or IgG immunoprecipitation, and the immunoprecipitates were analyzed by immunoblotting with the indicated antibodies. (D) Anti-Flag immunoprecipitates prepared from transfected 293FT cells were immunoblotted with the indicated antibodies. For anti-p-Tyr-phosphor-L-tyrosine blotting, anti-p-Tyr antibodies were incubated with the anti-phosphotyrosine inhibitor phospho-L-tyrosine for 8 h at 4°C before immunoblot analysis. (E) 293FT cells cotransfected with Flag-FOXM1 and Myc-ABL1 plasmids upon imatinib treatment for indicated time were analyzed by immunoprecipitation and immunoblotting. (F-H) Monophosphorylated peptides containing PO_3-modified tyrosine residues were identified by LC–MS/MS: LRPQTQTSY(p)DAK in F, Y(p)LTLDQVFK in G, and VSSY(p)LVPIQFPVNQS in H. (I) The relative p-Flag-FOXM1 levels from Fig.3E were quantified and statistically analyzed, and represented as mean ± SD of three independent analysis. ***$p<0.001$, ANOVA. (J) The reactivity and the specifictity of the anti-p-FOXM1Y575 antibody were verificated by ELISA. (K) FOXM1 WT, FOXM1Y575F proteins were incubated with recombinant ABL1 kinease domain protein at 30°C for 30 min in the presence of ATP. The products were analyzed by SDS–PAGE and immunoblotting with the indicated antibodies. (L) Anti-Flag immunoprecipitates prepared from 293FT cells coexpressing Flag-FOXM1 with Myc-ABL1 were immunoblotted with the indicated antibodies. The specificity of the anti-p-FOXM1Y575 antibody was shown in the middle panel by incubating the anti-p-FOXM1Y575 antibody with the antigen peptide Cys-PASQLSY(p)SQEVGG for 12 h at 4°C before immunoblot analysis. (M) 293FT cells cotransfected with Flag-FOXM1 and Myc-ABL1 plasmids were synchronized to prometaphase by 50 ng/ml nocodazole treatment for 12 h. Lysates were subjected to anti-Flag

immunoprecipitation and immunoblotted with the indicated antibodies. (N) HeLa cells treated with or without imatinib were subjected to anti-FOXM1 or IgG immunoprecipitation, and the immunoprecipitates were analyzed by immunoblotting with the indicated antibodies. (O) HeLa cells were incubated with primary anti-FOXM1 and anti-ABL1 antibodies, then incubated with FITC-linked and TRITC-linked secondary antibodies. Nuclei were stained with DAPI. The representative images were shown. (P) 293FT cells transfected with the indicated plasmids were analyzed by immunoprecipitation and immunoblotting.

Supplementary Fig.S4

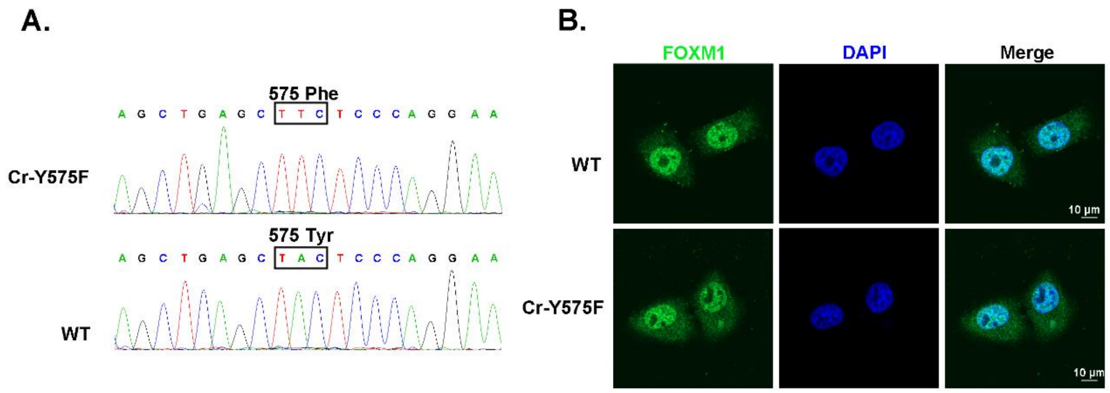

Supplementary Fig.S4 Cr-Y575F cell line verification.

(A) Genomic DNA was extracted from Cr-Y575F and wild type cells, and the target sequences of the region encoding FOXM1 Y575 were amplified and analyzed by sequencing. (B) Wild type or Cr-Y575F cells were incubated with primary anti-FOXM1 antibody and then incubated with FITC- linked secondary antibodies. Nuclei were stained with DAPI. The representative images were shown.

Supplementary Fig.S5

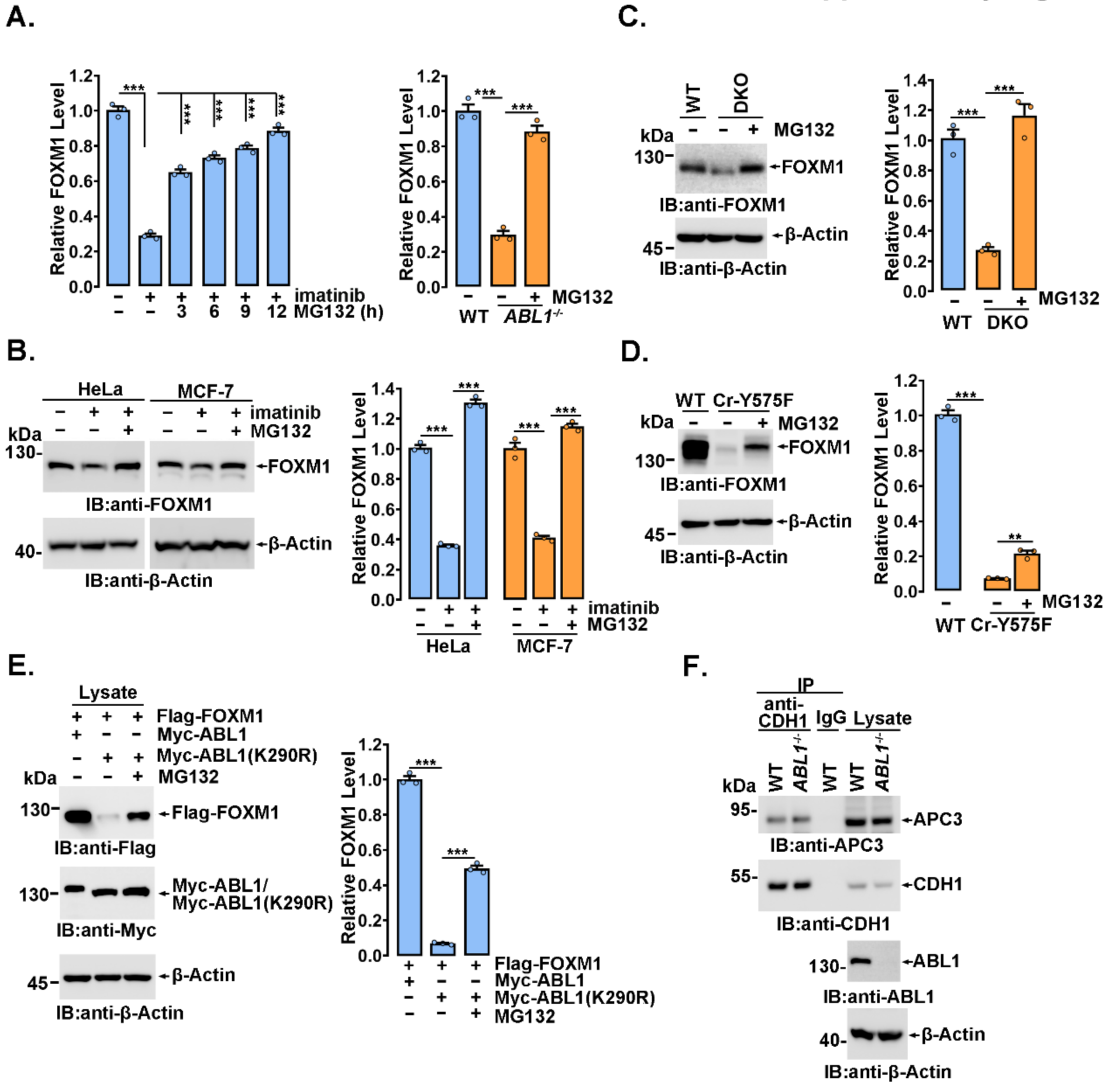

Supplementary Fig.S5 ABL1-mediated Y575 phosphorylation inhibits FOXM1 ubiquitination.

(A) The relative protein levels from Fig.5A were quantified and statistically analyzed, and represented as mean ± SD of three independent analysis. ***p<0.001, ANOVA. (B-D) HeLa or MCF-7 cells with the indicated imatinib or MG132 treatment were subjected to immunoblotting analysis (B). Lysates from wild-type and *abl1*$^{-/-}$*abl2*$^{-/-}$ MEFs treated with or without MG132 were analyzed by immunoblotting (C). Wild type or the Cr-Y575F cells treated with or without MG132 were subjected to immunoblotting analysis (D). (E) 293FT cells with the indicated treatment were analyzed by immunoblotting. The relative protein levels were quantified and

statistically analyzed, and represented as mean ± SD of three independent analysis. **$p<0.01$, ***$p<0.001$, ANOVA. (F) Wild type or *ABL1*$^{-/-}$ HeLa cells were subjected to anti-CDH1 or IgG immunoprecipitation, and the immunoprecipitates were analyzed by immunoblotting.

Supplementary Fig.S6

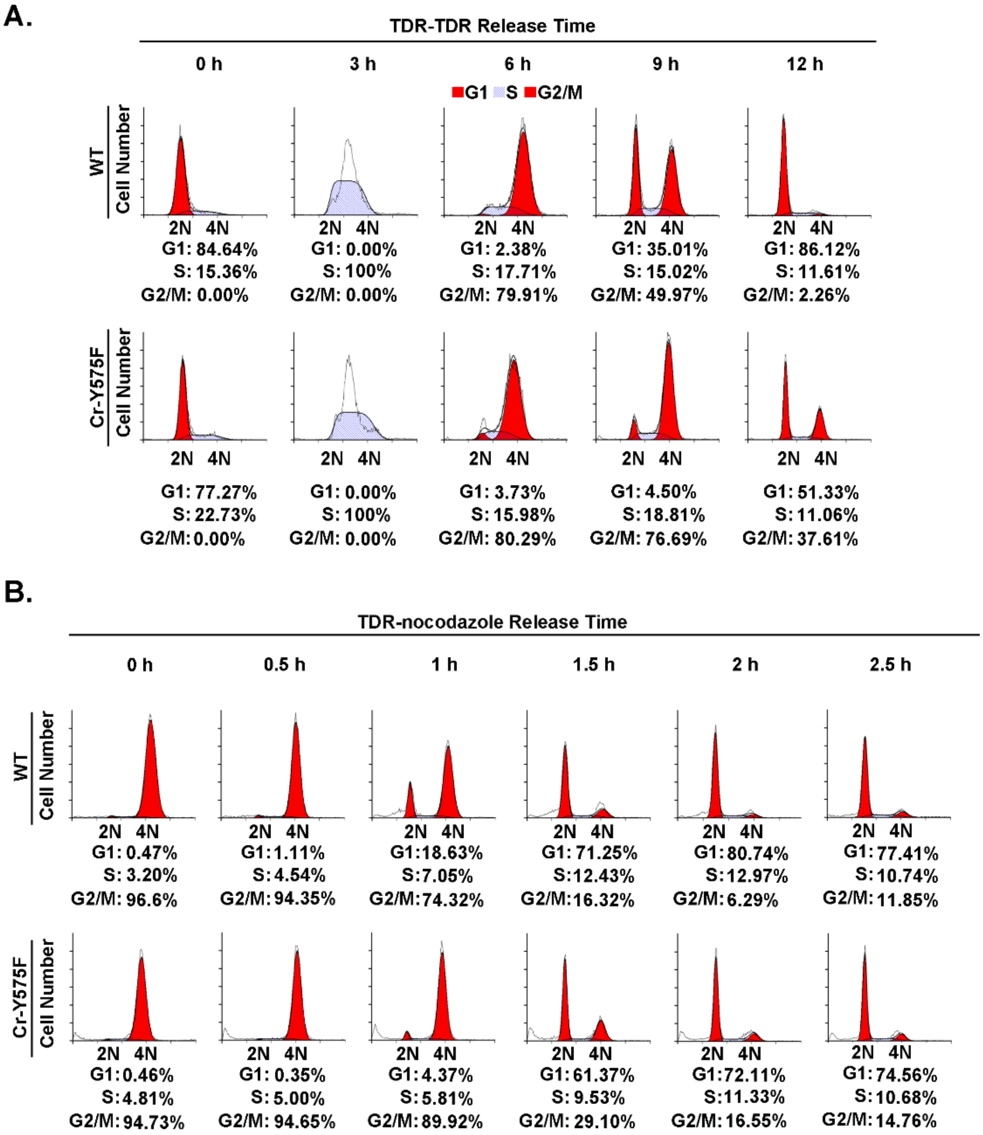

Supplementary Fig.S6

C.

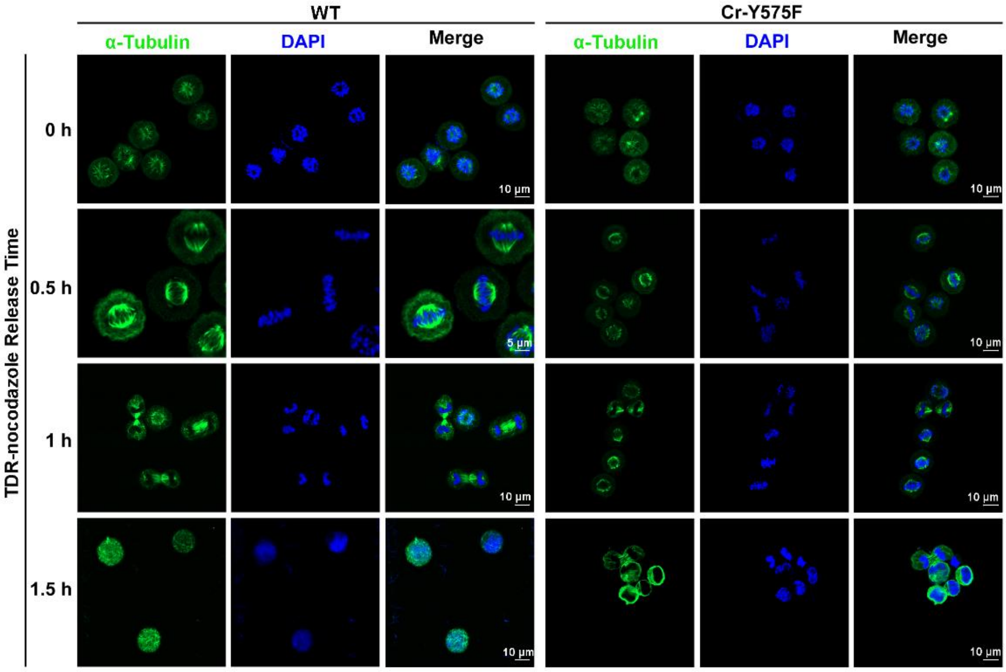

D.

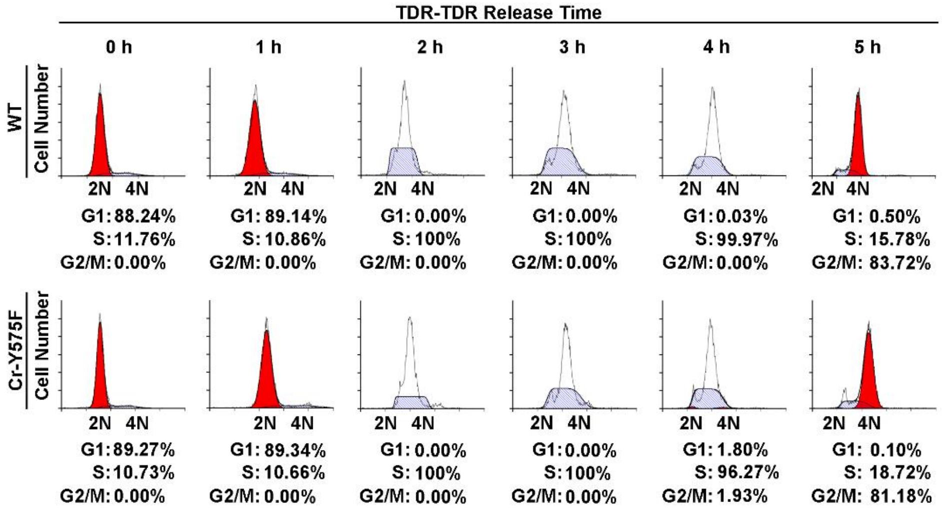

Supplementary Fig.S6 FOXM1 Y575 phosphorylation is critical for mitosis progression.

(A, B and D) Cr-Y575F and wild type cells were harvested at the indicated time points after double thymidine release (A and D) or thymidine-nocodazole

release (B), and the cell cycle was analyzed by flow cytometry. (C) The mitotic progression of thymidine-nocodazole synchronized cells was immunostained with an anti-α-tubulin antibody (green) and imaged by confocal microscopy.

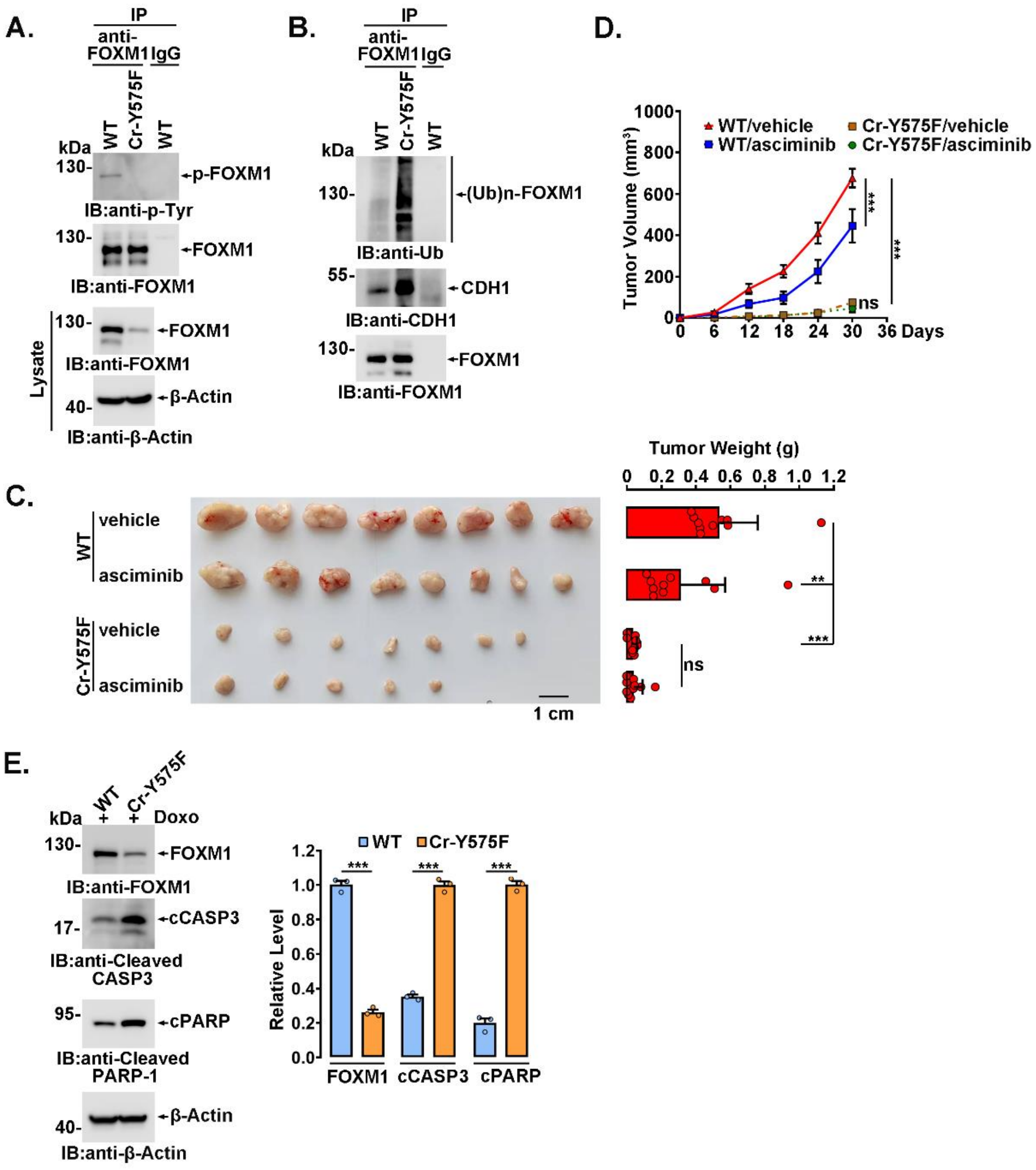

Supplementary Fig.S7 ABL1 mediated FOXM1 phosphorylation and stabilization contribute to tumor development.

(A and B) The MCF-7 Cr-Y575F and wild type cells were subjected to anti-FOXM1 or IgG immunoprecipitation, and the immunoprecipitates were

analyzed by immunoblotting.

(C and D) BALB/c null mice subcutaneously injected with MCF-7 Cr-Y575F and wild type cells were treated with or without the ABL1 inhibitor Asciminib (30 mg/kg) each day. The growth of the xenograft tumors were monitored (D). After 33 days, the tumors were dissected, then imaged and weighed (C). Data are presented as the means± SEM for eight mice per group. ns, not significant; **$p<0.01$, ***$p<0.001$, two-way ANOVA.

(E) Wild type or HeLa Cr-Y575F cells were treated with doxorubicin (3uM) and subjected to immunoblotting analysis with indicated antibodies. The relative proteins levels were quantified and statistically analyzed, and represented as mean ± SD of three independent analysis. ***$p<0.001$, ANOVA.

Supplementary Fig.S8

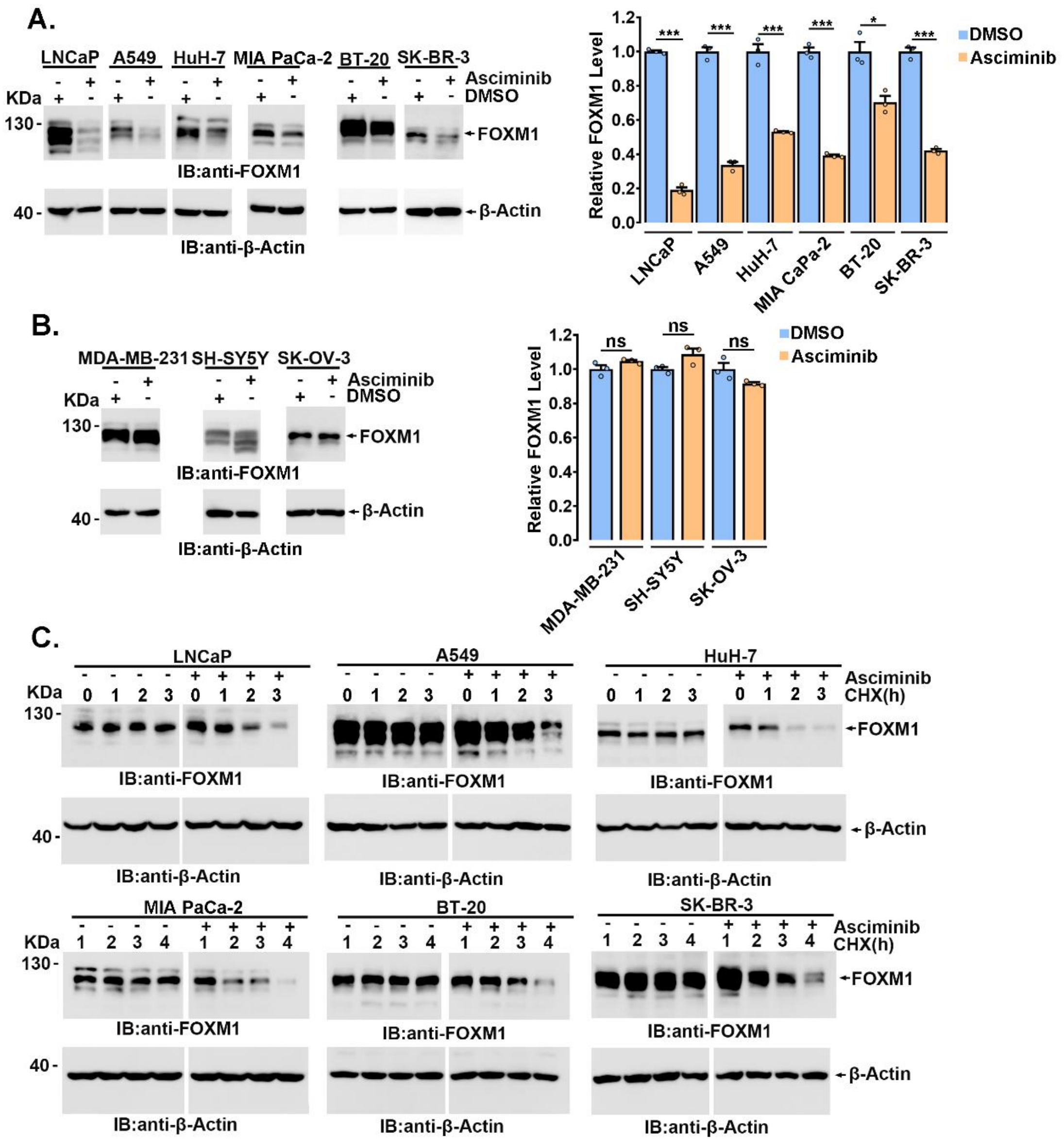

Supplementary Fig.S8 Asciminib treatment decreased FOXM1 expression and impaired protein stability in cancer cell lines.

(A and B) The indicated cancer cell lines treated with asciminib (20 μM, 18 h) or vehicle were subjected to immunoblotting analysis. (C) The indicated cells

were treated with 100 μg/ml cycloheximide (CHX) after subjected to 20 μM asciminib or vehicle treatment for one hours, and the cells were harvested at the indicated hours and analyzed by immunoblotting. Data shown represent the means±SD of biological triplicates. *$p<0.05$, ***$p<0.001$, ns not significant, Student's t test.

Supplementary Table 1

qRT–PCR primers used for wild-type and *abl1*$^{-/-}$*abl2*$^{-/-}$ MEF analysis:

FOXM1-F: 5’-GTCTCCTTCTGGACCATTCACC-3’
FOXM1-R: 5’-GCTCAGGATTGGGTCGTTTCTG-3’
Plk1-F: 5’-CCATCTTCTGGGTCAGCAAGTG-3’
Plk1-R: 5’- CCGTCATTGTAGAGAATCAGGCG-3’
Aurora B-F: 5’-AGGTCTGCAGGGAGAACTGA-3’
Aurora B-R: 5’-AGGCACAGAAGAGGGGAACT-3’
Cyclin B1-F: 5’- CTGACCCAAACCTCTGTAGTG-3’
Cyclin B1-R: 5’-CCTGTATTAGCCAGTCAATGAGG-3’
Cdc25B-F: 5'-ATTCTCGTCTGAGCGTGGAC-3’
Cdc25B-R: 5'-GTTCGGATGCTGTGGGAAG-3’
GAPDH-F: 5’-CATCACTGCCACCCAGAAGACTG-3’
GAPDH-R: 5’-ATGCCAGTGAGCTTCCCGTTCAG-3’

Supplementary Table 2

qRT–PCR primers used for control and Cr-Y575F cell analysis:

CENP A-F: 5’-CTT CCT CCC ATC AAC ACA GTC G-3’
CENP A-R: 5’-TGC TTC TGC TGC CTC TTG TAG G-3’
CKS2-F: 5’-GCTCTTCGCGCTCTCGTTTCATTT -3’
CKS2-R: 5’-ACTCTGTTGGACACCAAGTCTCCT-3’
CENP E-F: 5’-TAACACGGATGCTGGTGACCTCTT -3’
CENP E-R: 5’-TGCCAAGGCACCAAGTAACTCTTC-3’
PLK1-F: 5’-AACGACTTCGTGTTCGTGGTGTTG-3’
PLK1-R: 5’-GGTACTGGCAGCCAAGCACAATTT-3’
CYCLIN B1-F: 5’-TTTGCACTTCCTTCGGAGAGC-3’
CYCLIN B1-R: 5’-AAGGAGGAAAGTGCACCATGTC-3’
β-actin-F: 5’-TGGCACCCAGCACAATGAA-3’
β-actin-R: 5’-CTAAGTCATAGTCCGCCTAGAAGCA-3’

Supplementary Table 3

sgRNA sequence to generate the *abl1* knockout cell line:

ABL1-sgRNA: 5’-TGTGATTATAGCCTAAGACCCGG-3’

Supplementary Table 4

Sequence to generate the Cr-Y575F cell line:

FOXM1(Y575F) -sgRNA: 5’-CTCCCAGCTCAGCTACTCCC-3’
FOXM1Y575- oligo donor: 5’-CGCTGGGCCGCAGAGCTCCCGTTCCCAGCAGACTCCTCTGACCCTGCCTCCCAGCTGAGCTTCTCCCAGGAAGTGGGAGGACCTTTTAAGACACCCATTAAGGAAACGCTGCCCATCTCCTCC-3’
