# Supplementary material for: ABL1-mediated phosphorylation promotes FOXM1-related tumorigenicity by Increasing FOXM1 stability
Source: Cell Death Differ. 2024 Jul 26;31(10):1285–301. doi: 10.1038/s41418-024-01339-w (PMC11445503; doi:10.1038/s41418-024-01339-w)

Fig.1

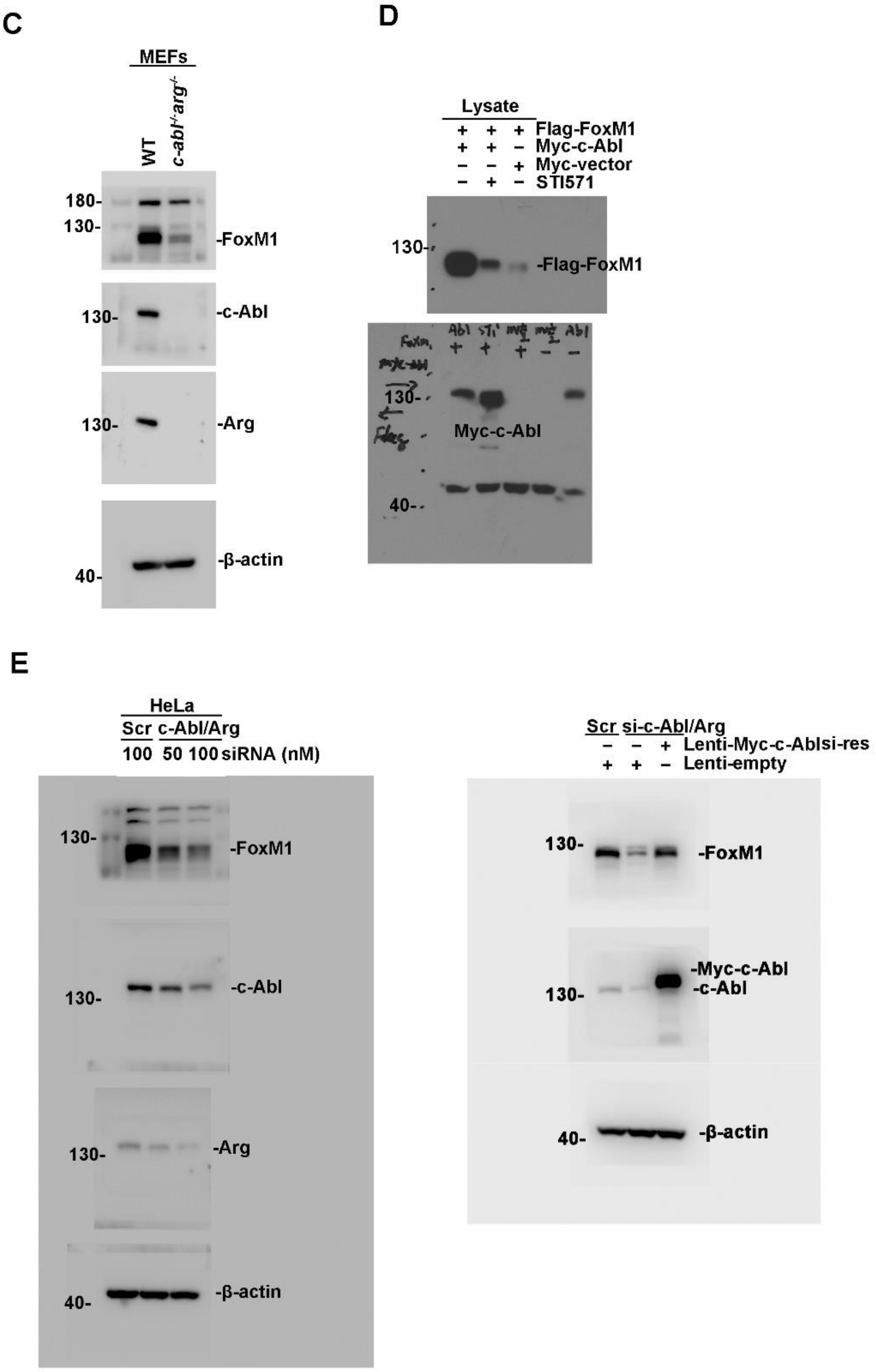
C
MEFs
WT
c-abl-/-arg-/-
180-
130-
-FoxM1
130-
-c-Abl
130-
-Arg
40-
-β-actin
D
Lysate
+ + + Flag-FoxM1
+ + − Myc-c-Abl
− − + Myc-vector
− + − STI571
130-
-Flag-FoxM1
130-
Myc-c-Abl
40-
E
HeLa
Scr c-Abl/Arg
100 50 100 siRNA (nM)
130-
-FoxM1
130-
-c-Abl
130-
-Arg
40-
-β-actin
Scr si-c-Abl/Arg
− − + Lenti-Myc-c-Ablsi-res
+ + − Lenti-empty
130-
-FoxM1
-Myc-c-Abl
-c-Abl
130-
40-
-β-actin

Fig.1

F

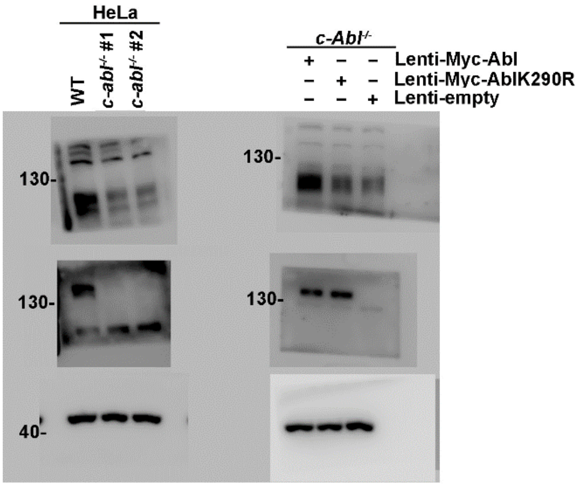
HeLa
WT
c-abl-/- #1
c-abl-/- #2
c-Abl-/-
Lenti-Myc-Abl
Lenti-Myc-AblK290R
Lenti-empty
130-
130-
40-
130-
130-

G

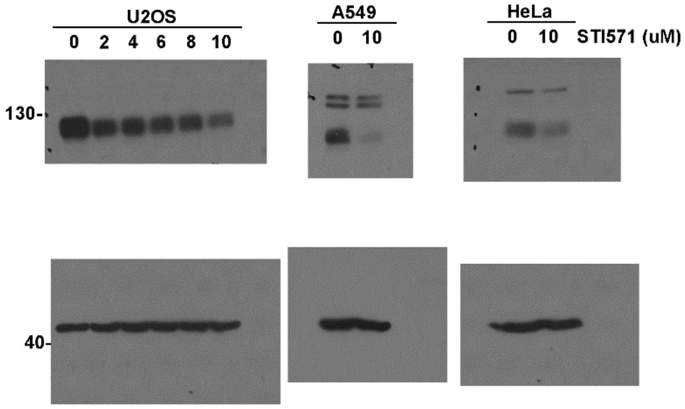
U2OS
0 2 4 6 8 10
A549
0 10
HeLa
0 10
STI571 (uM)
130-
40-

Fig.2

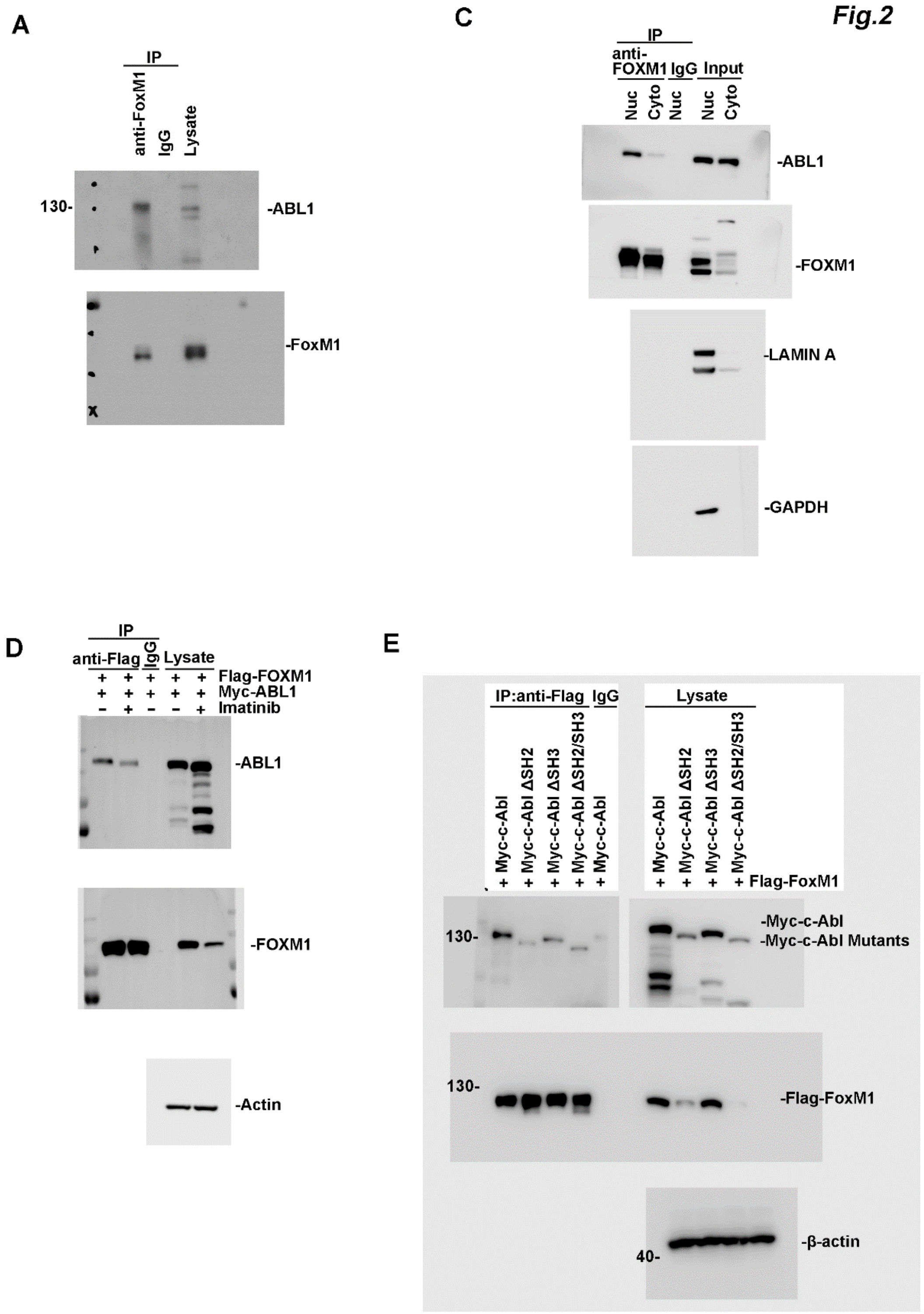
A
IP
anti-FoxM1
IgG
Lysate
130-
-ABL1
-FoxM1
C
IP
anti-FOXM1
IgG
Input
Nuc
Cyto
Nuc
Nuc
Cyto
-ABL1
-FOXM1
-LAMIN A
-GAPDH
D
IP
anti-Flag
IgG
Lysate
Flag-FOXM1
Myc-ABL1
Imatinib
-ABL1
-FOXM1
-Actin
E
IP:anti-Flag
IgG
Lysate
Myc-c-Abl
Myc-c-Abl ΔSH2
Myc-c-Abl ΔSH3
Myc-c-Abl ΔSH2/SH3
Myc-c-Abl
Flag-FoxM1
130-
-Myc-c-Abl
-Myc-c-Abl Mutants
-Flag-FoxM1
40-
-β-actin

Fig.2

F

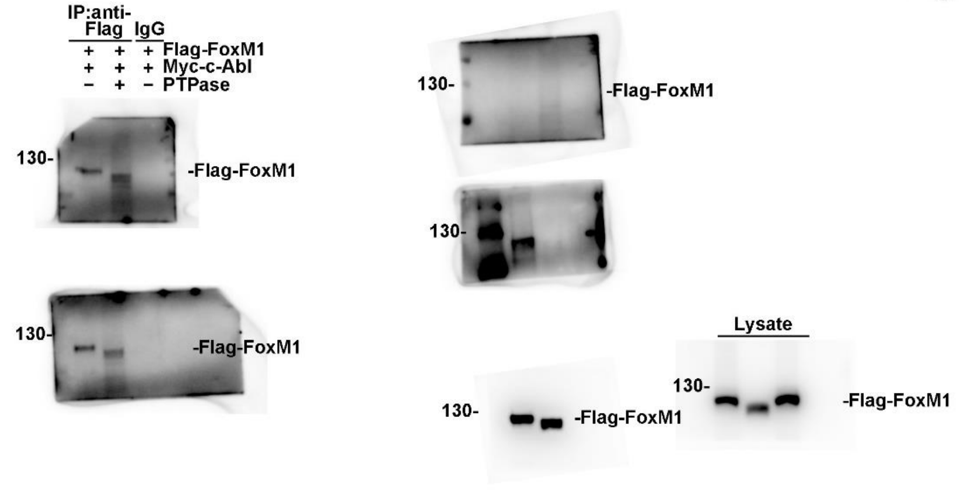
IP:anti-Flag
IgG
Flag-FoxM1
Myc-c-Abl
PTPase
130-
-Flag-FoxM1
Lysate

G

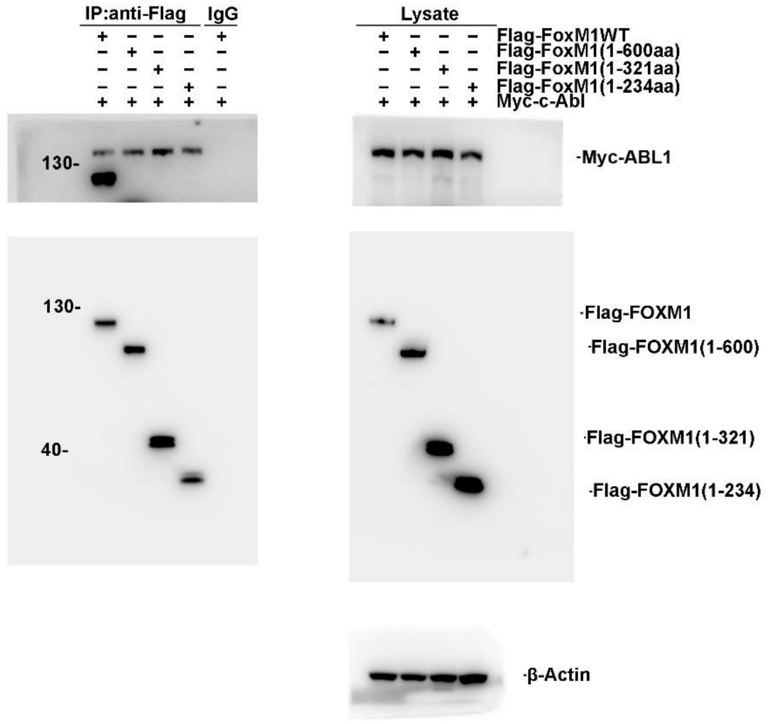
IP:anti-Flag
IgG
Lysate
Flag-FoxM1WT
Flag-FoxM1(1-600aa)
Flag-FoxM1(1-321aa)
Flag-FoxM1(1-234aa)
Myc-c-Abl
130-
40-
-Myc-ABL1
-Flag-FOXM1
-Flag-FOXM1(1-600)
-Flag-FOXM1(1-321)
-Flag-FOXM1(1-234)
-β-Actin

Fig.3

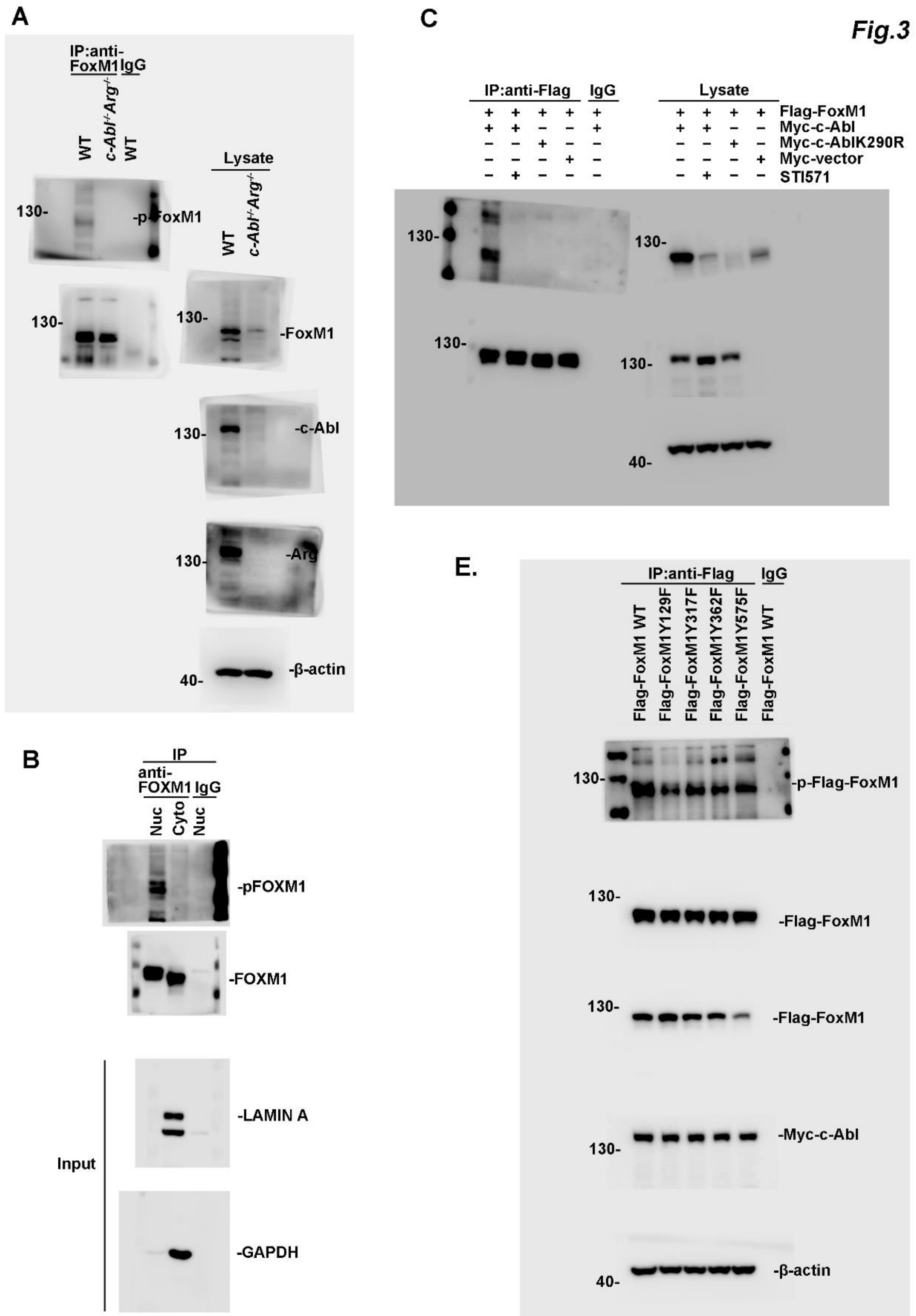

A
IP:anti-FoxM1
IgG
WT
c-Abl-/-Arg-/-
WT
Lysate
WT
c-Abl-/-Arg-/-
130-
-p-FoxM1
130-
-FoxM1
130-
-c-Abl
130-
-Arg
40-
-β-actin
B
IP
anti-FOXM1
IgG
Nuc
Cyto
Nuc
-pFOXM1
-FOXM1
Input
-LAMIN A
-GAPDH
C
IP:anti-Flag
IgG
Lysate
Flag-FoxM1
Myc-c-Abl
Myc-c-AblK290R
Myc-vector
STI571
130-
130-
130-
130-
40-
E.
IP:anti-Flag
IgG
Flag-FoxM1 WT
Flag-FoxM1Y129F
Flag-FoxM1Y317F
Flag-FoxM1Y362F
Flag-FoxM1Y575F
Flag-FoxM1 WT
130-
-p-Flag-FoxM1
130-
-Flag-FoxM1
130-
-Flag-FoxM1
130-
-Myc-c-Abl
40-
-β-actin

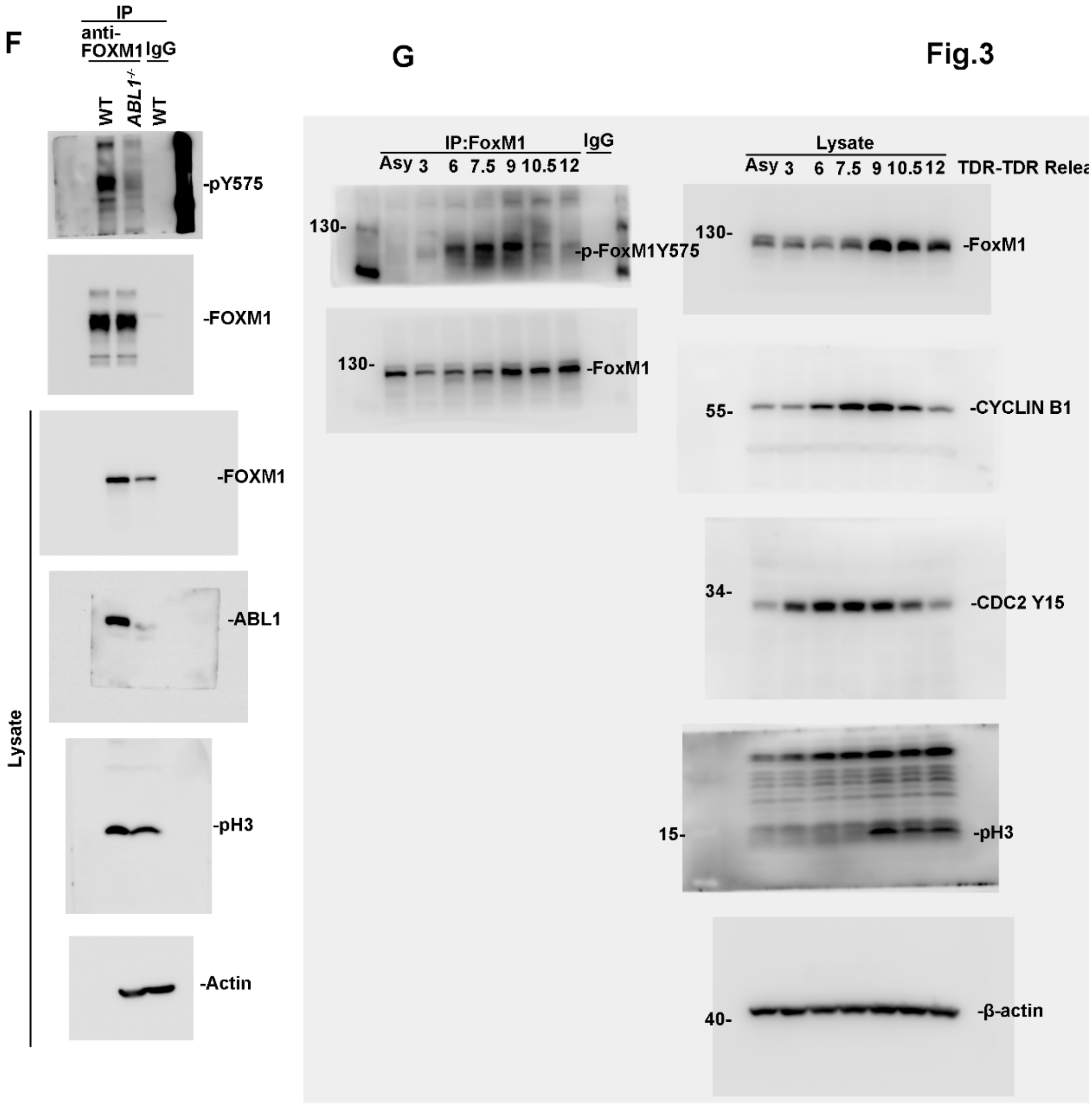
F
IP
anti-FOXM1
IgG
WT
ABL1-/-
WT
-pY575
-FOXM1
-FOXM1
-ABL1
-pH3
-Actin
Lysate
G
IP:FoxM1
IgG
Asy 3 6 7.5 9 10.5 12
130-
-p-FoxM1Y575
130-
-FoxM1
Lysate
Asy 3 6 7.5 9 10.5 12
TDR-TDR Relea
130-
-FoxM1
55-
-CYCLIN B1
34-
-CDC2 Y15
15-
-pH3
40-
-β-actin

Fig.3

Fig.4

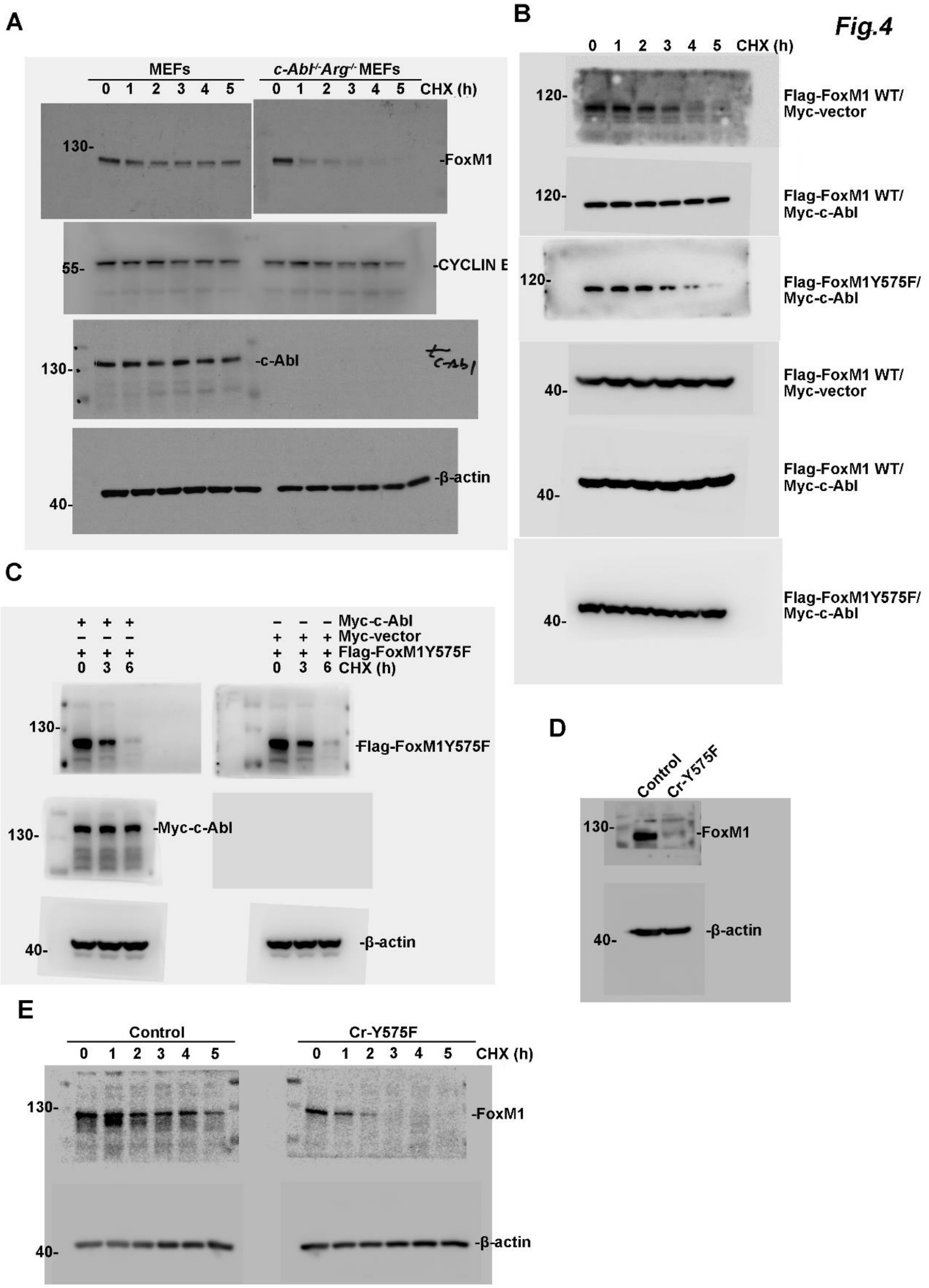
A
MEFs
c-Abl-/-Arg-/- MEFs
0 1 2 3 4 5 0 1 2 3 4 5 CHX (h)
130-
-FoxM1
55-
-CYCLIN E
130-
-c-Abl
40-
-β-actin
B
0 1 2 3 4 5 CHX (h)
120-
Flag-FoxM1 WT/ Myc-vector
120-
Flag-FoxM1 WT/ Myc-c-Abl
120-
Flag-FoxM1Y575F/ Myc-c-Abl
40-
Flag-FoxM1 WT/ Myc-vector
40-
Flag-FoxM1 WT/ Myc-c-Abl
40-
Flag-FoxM1Y575F/ Myc-c-Abl
C
+ + + − − − Myc-c-Abl
− − − + + + Myc-vector
+ + + + + + Flag-FoxM1Y575F
0 3 6 0 3 6 CHX (h)
130-
-Flag-FoxM1Y575F
130-
-Myc-c-Abl
40-
-β-actin
D
Control
Cr-Y575F
130-
-FoxM1
40-
-β-actin
E
Control
Cr-Y575F
0 1 2 3 4 5 0 1 2 3 4 5 CHX (h)
130-
-FoxM1
40-
-β-actin

Fig.5

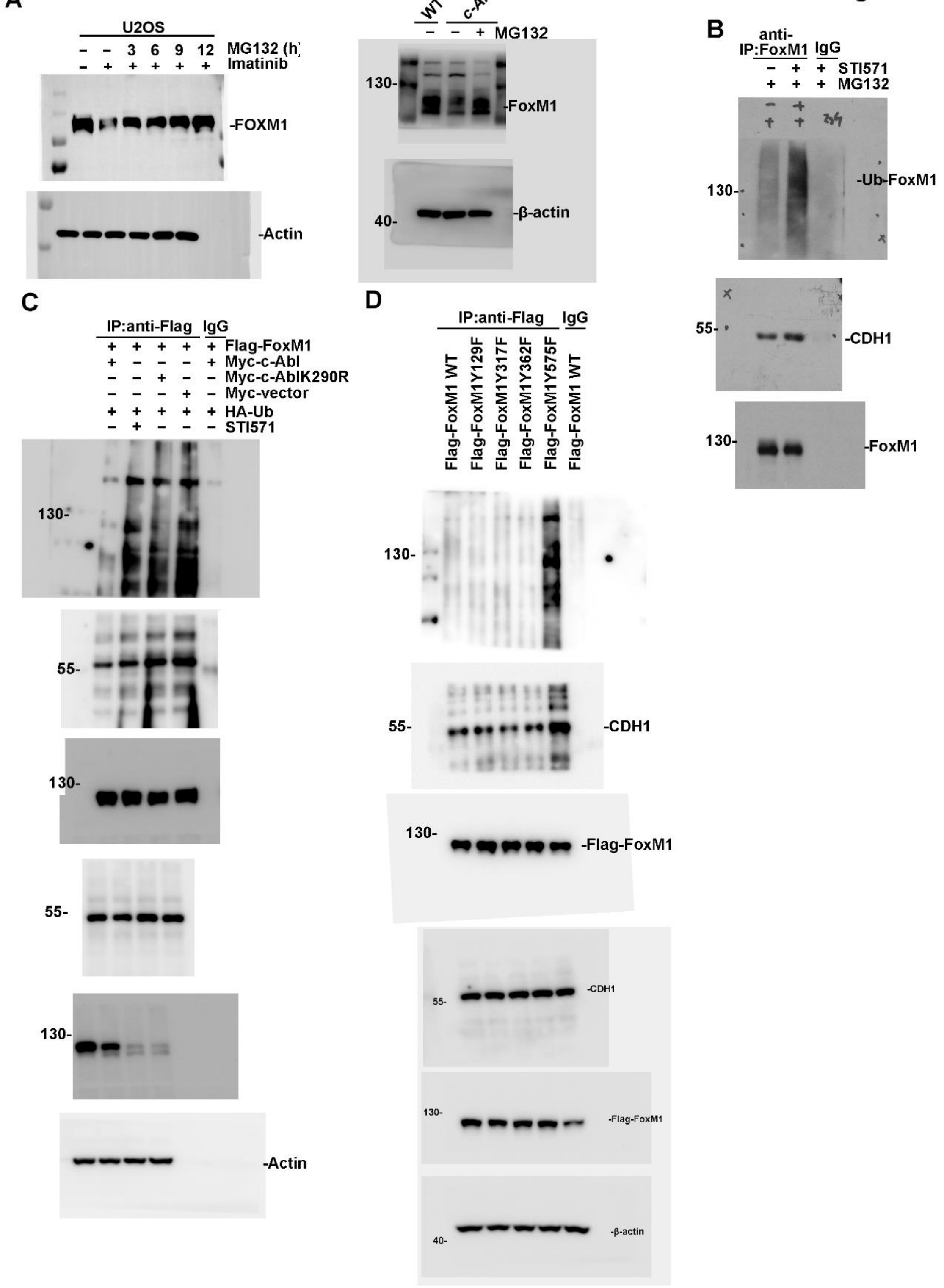

A
U2OS
MG132 (h)
Imatinib
– – 3 6 9 12
– + + + + +
-FOXM1
-Actin
WT
c-Abl-/-
– – + MG132
130-
-FoxM1
40-
-β-actin
B
anti-
IP:FoxM1
IgG
– + + STI571
+ + + MG132
130-
-Ub-FoxM1
55-
-CDH1
130-
-FoxM1
C
IP:anti-Flag
IgG
Flag-FoxM1
Myc-c-Abl
Myc-c-AblK290R
Myc-vector
HA-Ub
STI571
130-
55-
130-
55-
130-
-Actin
D
IP:anti-Flag
IgG
Flag-FoxM1 WT
Flag-FoxM1Y129F
Flag-FoxM1Y317F
Flag-FoxM1Y362F
Flag-FoxM1Y575F
Flag-FoxM1 WT
130-
55-
-CDH1
130-
-Flag-FoxM1
-CDH1
55-
130-
-Flag-FoxM1
40-
-β-actin

Fig.5

E

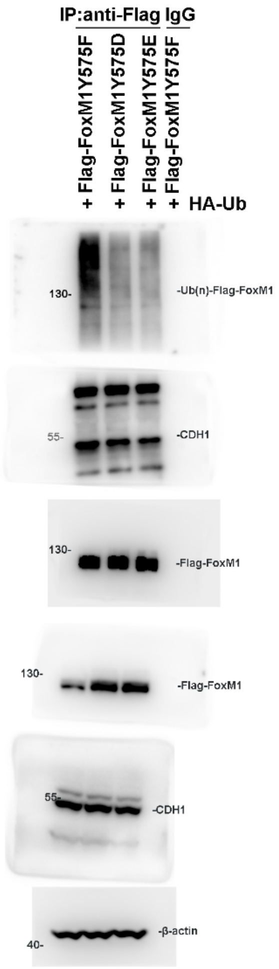
IP:anti-Flag IgG
Flag-FoxM1Y575F
Flag-FoxM1Y575D
Flag-FoxM1Y575E
Flag-FoxM1Y575F
HA-Ub
130-
-Ub(n)-Flag-FoxM1
55-
-CDH1
130-
-Flag-FoxM1
130-
-Flag-FoxM1
55-
-CDH1
40-
-β-actin

F

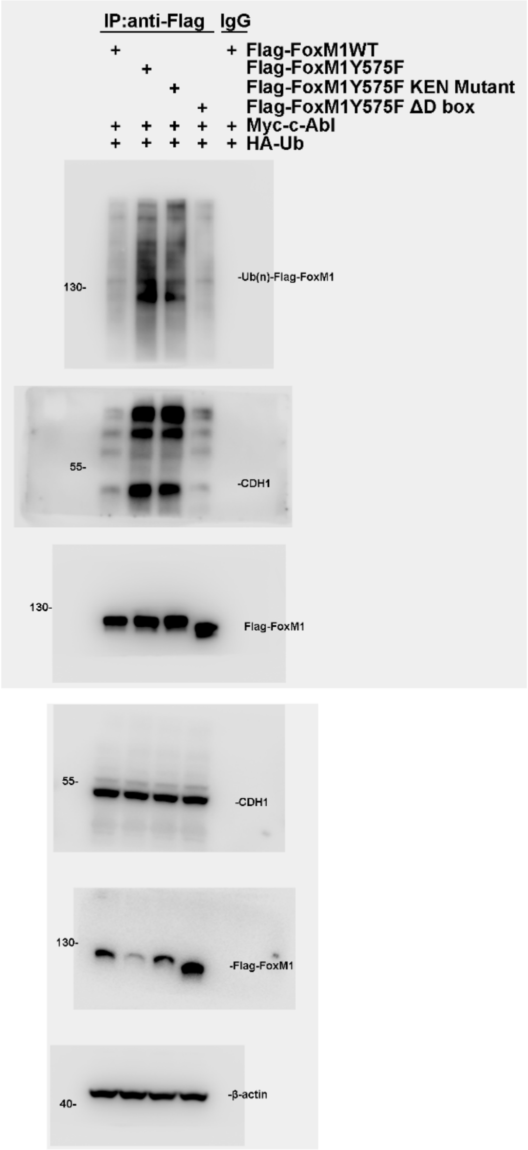
IP:anti-Flag IgG
Flag-FoxM1WT
Flag-FoxM1Y575F
Flag-FoxM1Y575F KEN Mutant
Flag-FoxM1Y575F ΔD box
Myc-c-Abl
HA-Ub
130-
-Ub(n)-Flag-FoxM1
55-
-CDH1
130-
Flag-FoxM1
55-
-CDH1
130-
-Flag-FoxM1
40-
-β-actin

Fig.5

G

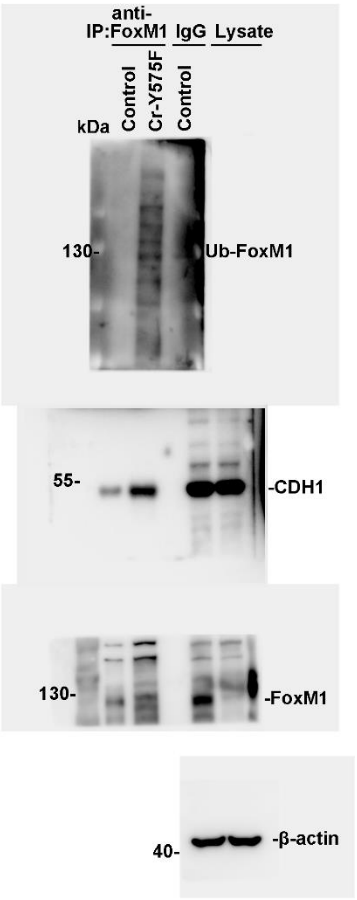

anti-
IP:FoxM1
IgG
Lysate
Control
Cr-Y575F
Control
kDa
130-
Ub-FoxM1
55-
-CDH1
130-
-FoxM1
40-
-β-actin

H

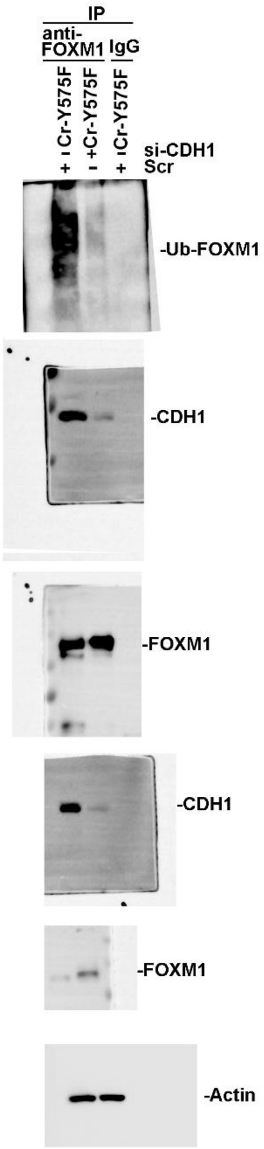

IP
anti-
FOXM1
IgG
Cr-Y575F
Cr-Y575F
Cr-Y575F
si-CDH1
Scr
-Ub-FOXM1
-CDH1
-FOXM1
-CDH1
-FOXM1
-Actin

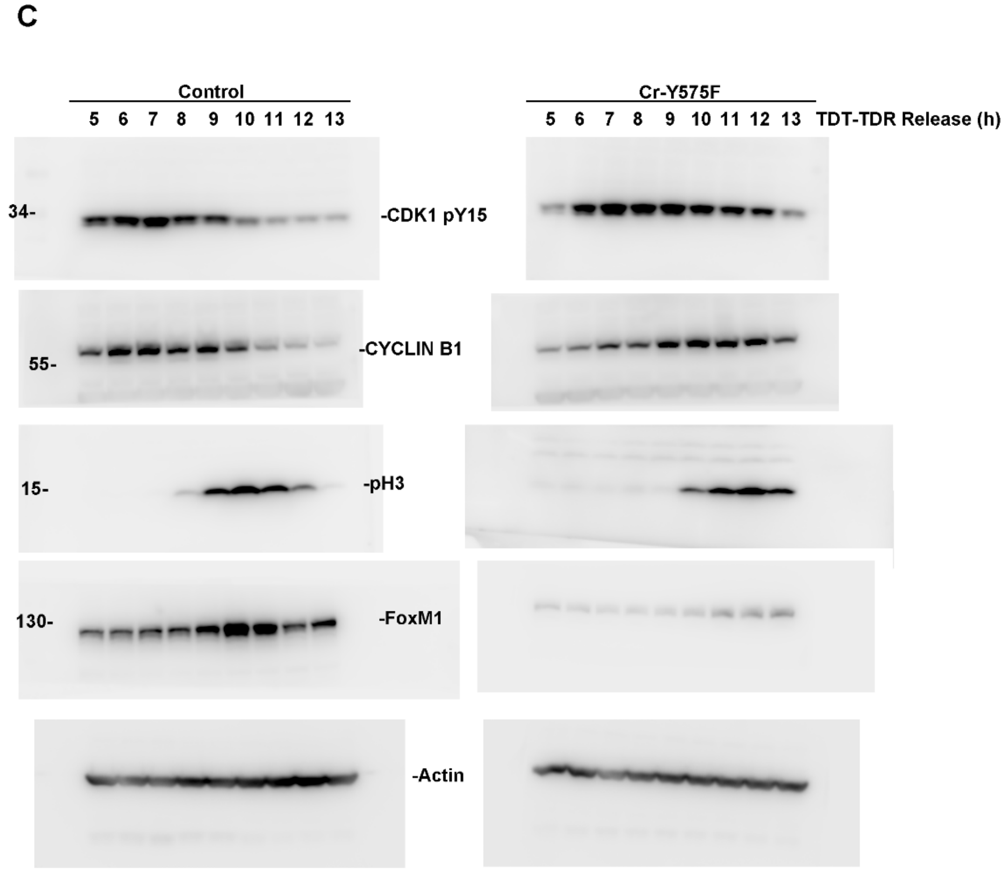
C
Control
Cr-Y575F
5 6 7 8 9 10 11 12 13
5 6 7 8 9 10 11 12 13 TDT-TDR Release (h)
34-
-CDK1 pY15
55-
-CYCLIN B1
15-
-pH3
130-
-FoxM1
-Actin

Supplementary Fig.S1

C

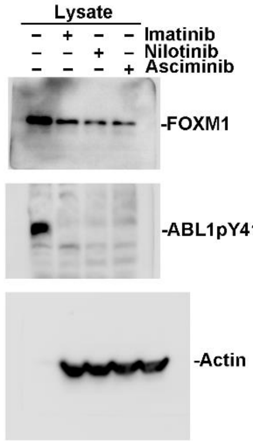

D-E

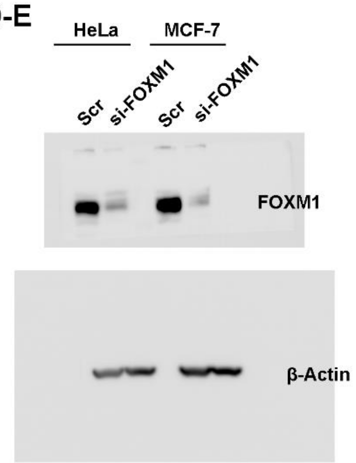

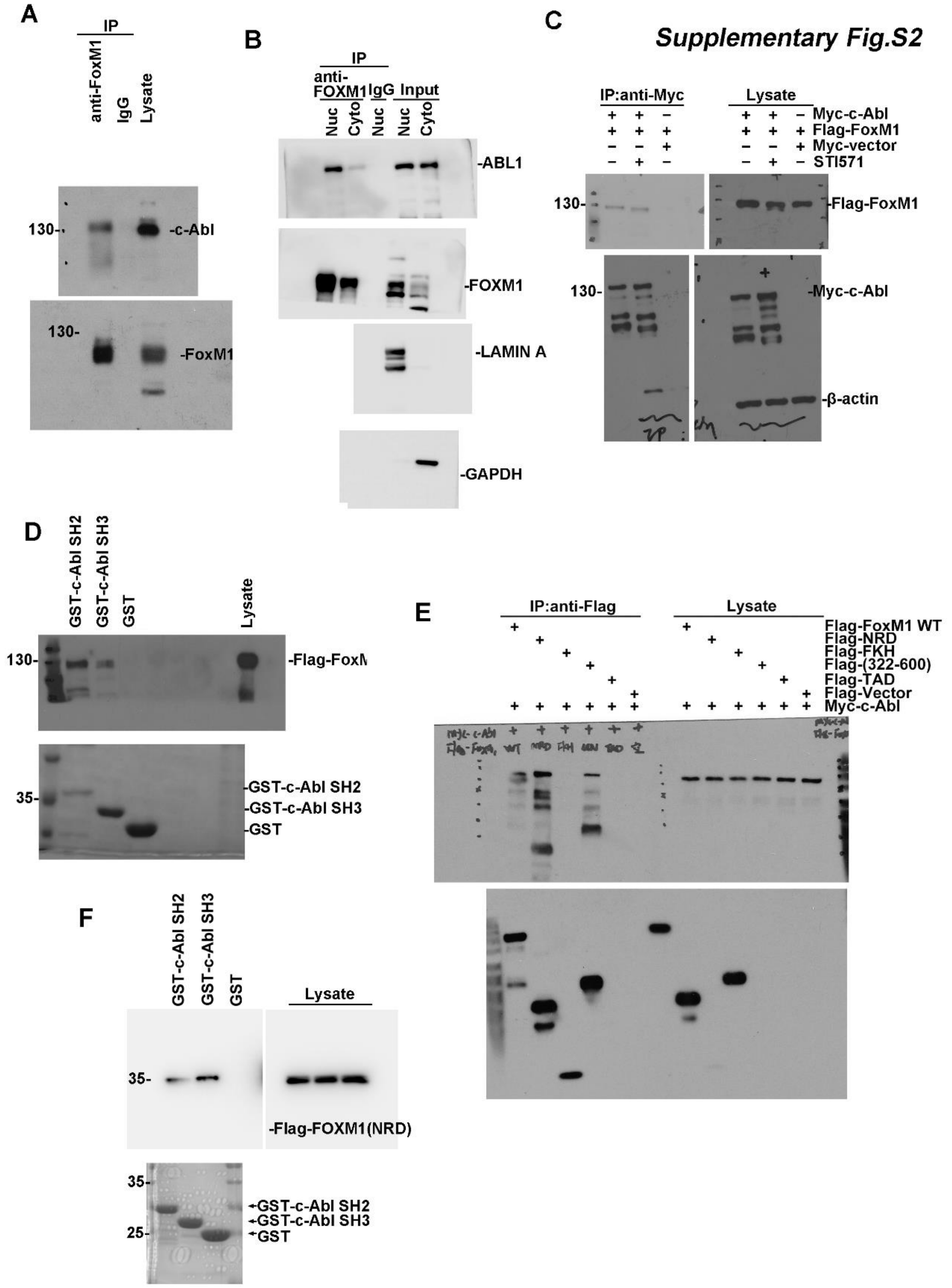

Supplementary Fig.S2
A
IP
anti-FoxM1
IgG
Lysate
130-
-c-Abl
130-
-FoxM1
B
IP
anti-FOXM1
IgG
Input
Nuc
Cyto
Nuc
Nuc
Cyto
-ABL1
-FOXM1
-LAMIN A
-GAPDH
C
IP:anti-Myc
Lysate
Myc-c-Abl
Flag-FoxM1
Myc-vector
STI571
130-
-Flag-FoxM1
130-
-Myc-c-Abl
-β-actin
D
GST-c-Abl SH2
GST-c-Abl SH3
GST
Lysate
130-
-Flag-FoxM
35-
-GST-c-Abl SH2
-GST-c-Abl SH3
-GST
E
IP:anti-Flag
Lysate
Flag-FoxM1 WT
Flag-NRD
Flag-FKH
Flag-(322-600)
Flag-TAD
Flag-Vector
Myc-c-Abl
F
GST-c-Abl SH2
GST-c-Abl SH3
GST
Lysate
35-
-Flag-FOXM1(NRD)
35-
25-
GST-c-Abl SH2
GST-c-Abl SH3
GST

Supplementary Fig.S3

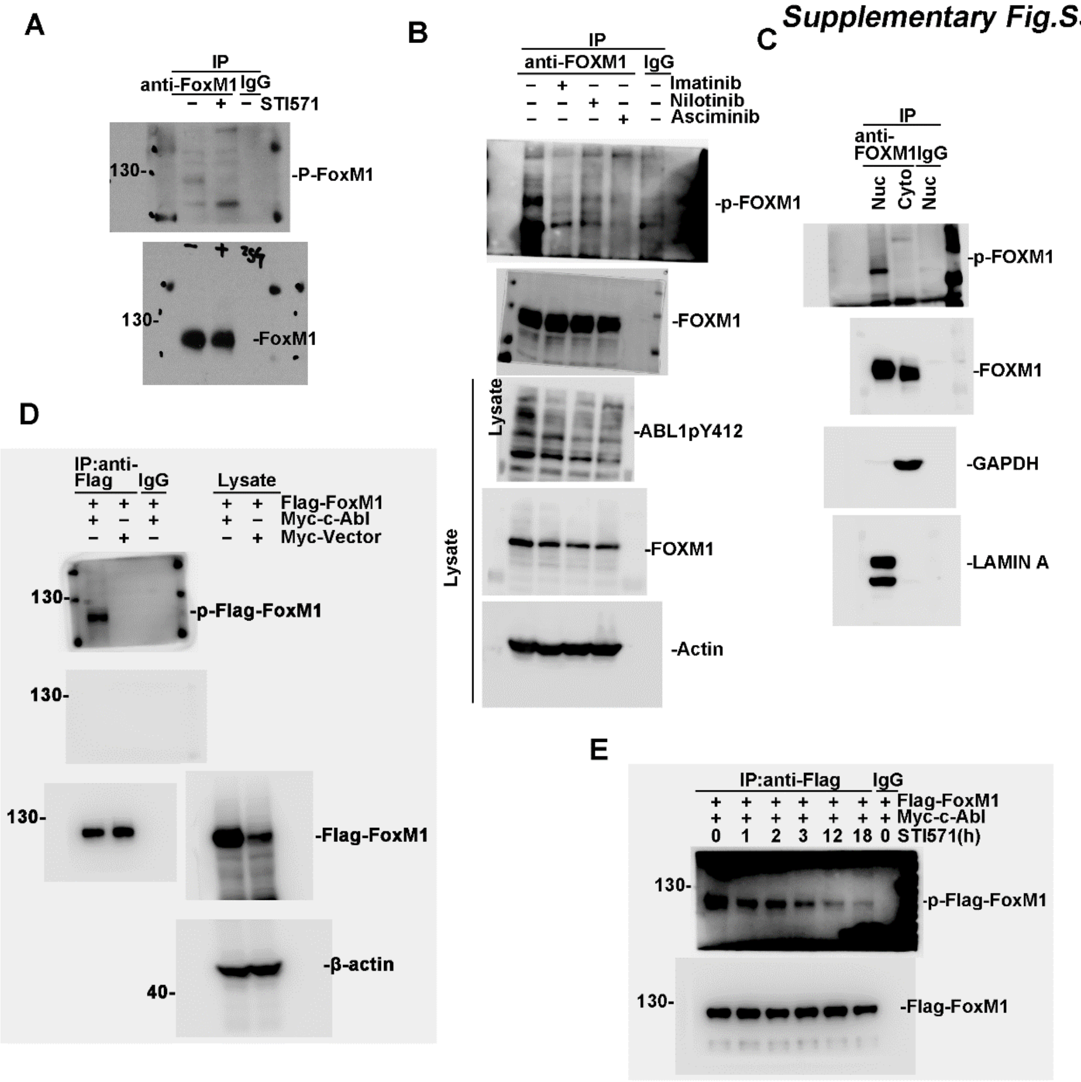

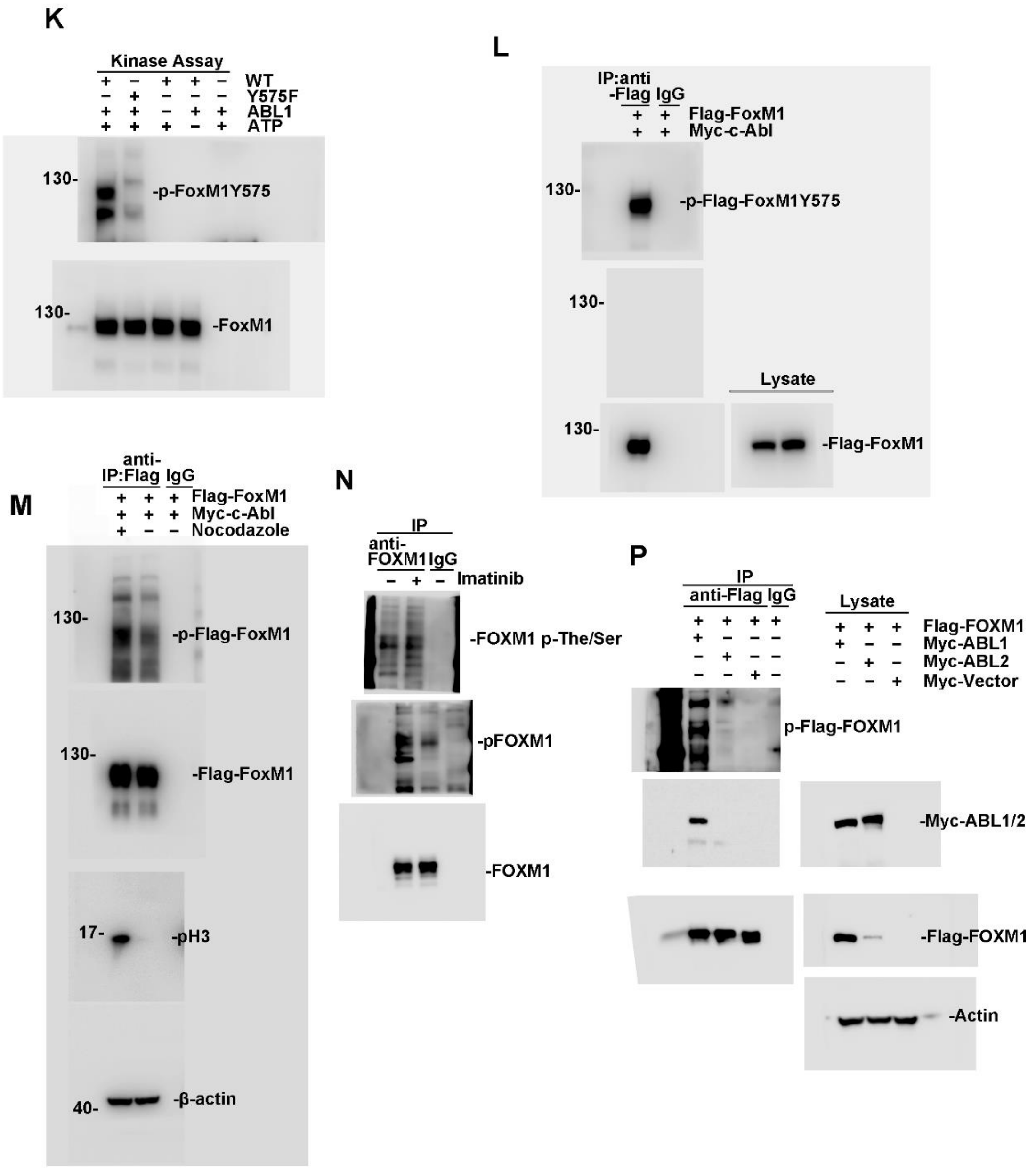
K
Kinase Assay
WT
Y575F
ABL1
ATP
130-
-p-FoxM1Y575
130-
-FoxM1
L
IP:anti -Flag
IgG
Flag-FoxM1
Myc-c-Abl
130-
-p-Flag-FoxM1Y575
130-
Lysate
130-
-Flag-FoxM1
M
anti-IP:Flag
IgG
Flag-FoxM1
Myc-c-Abl
Nocodazole
130-
-p-Flag-FoxM1
130-
-Flag-FoxM1
17-
-pH3
40-
-β-actin
N
IP
anti-FOXM1
IgG
Imatinib
-FOXM1 p-The/Ser
-pFOXM1
-FOXM1
P
IP
anti-Flag
IgG
Lysate
Flag-FOXM1
Myc-ABL1
Myc-ABL2
Myc-Vector
p-Flag-FOXM1
-Myc-ABL1/2
-Flag-FOXM1
-Actin

Supplementary Fig.S5

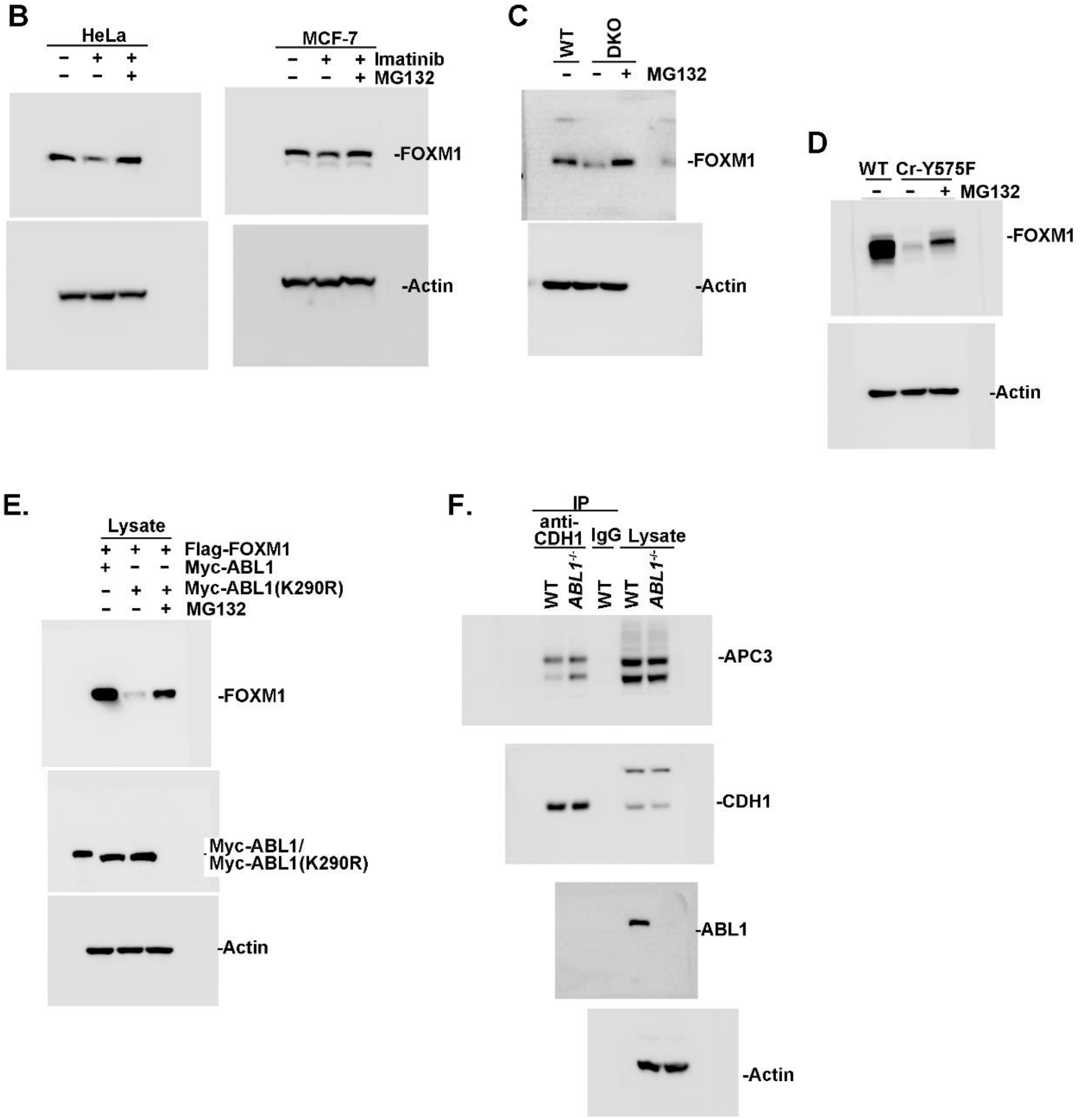

Supplementary Fig.S7

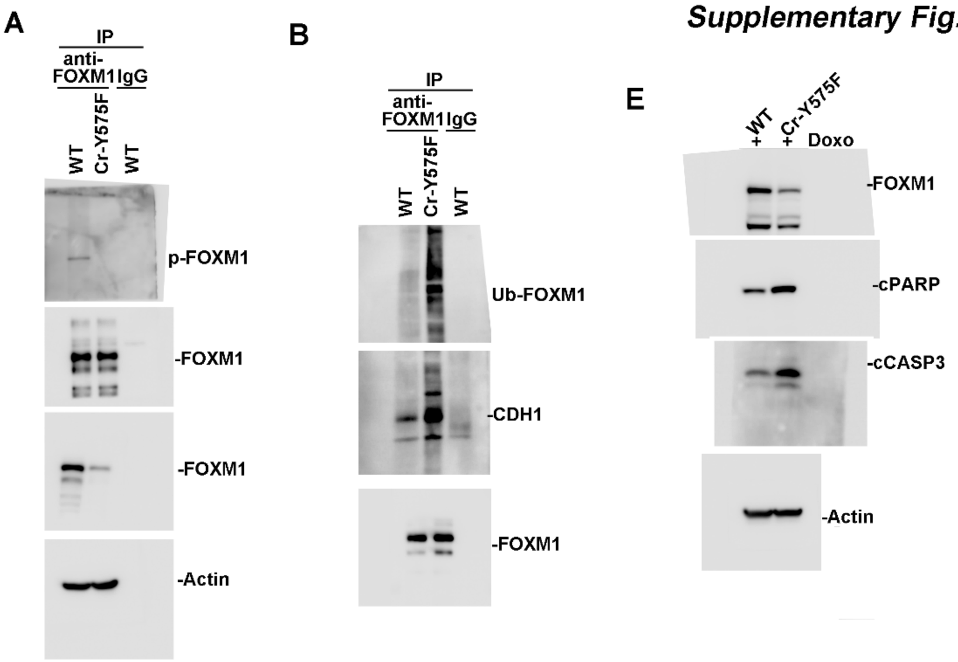

Supplementary Fig.S8

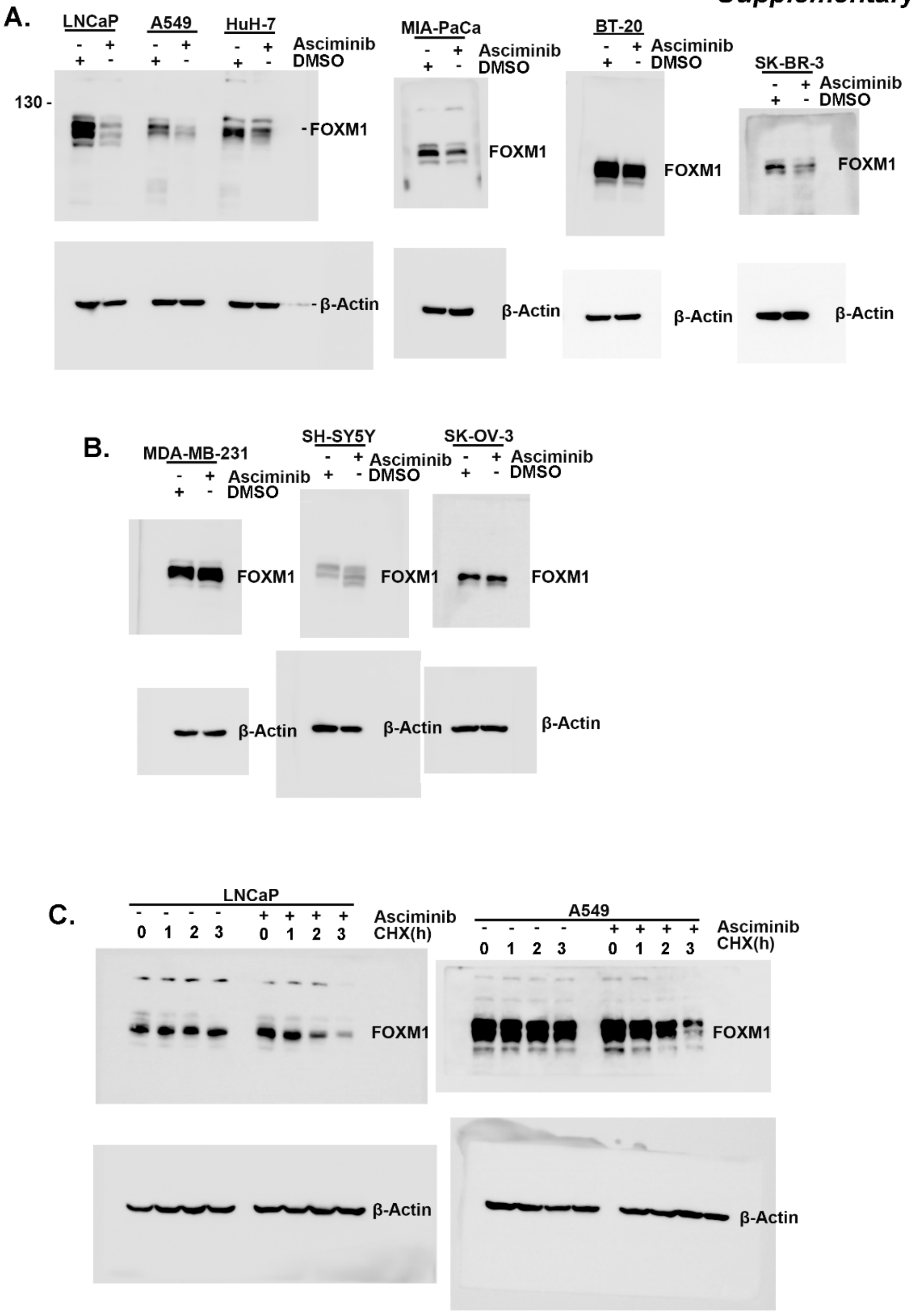

Supplementary Fig.S8

C.

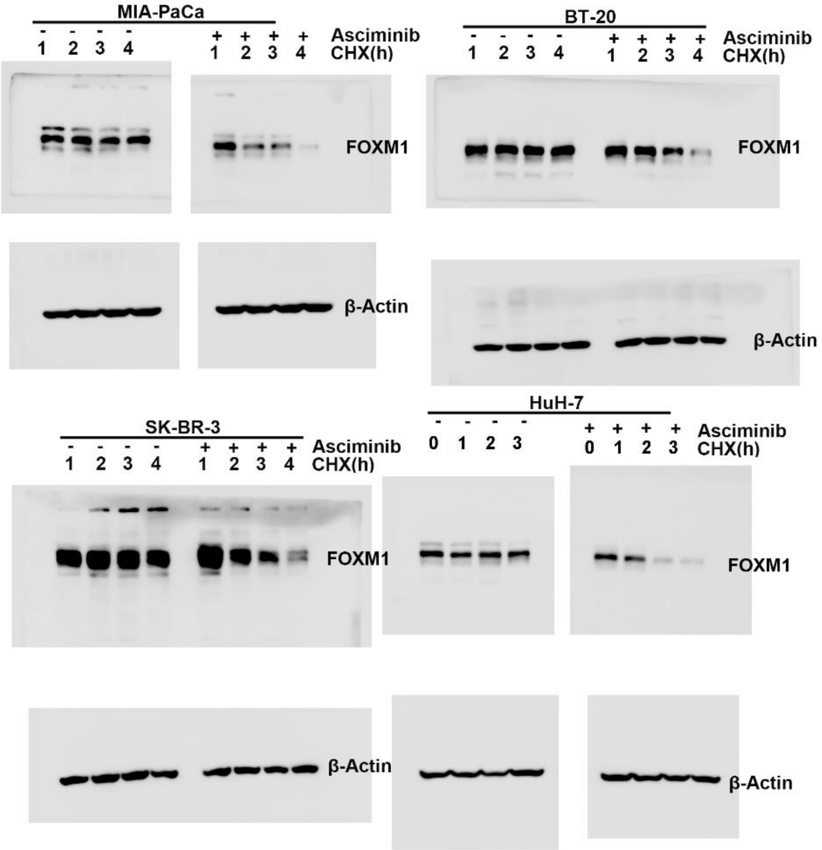

Supplement: Supplementary file 2 — original western blots [file 41418_2024_1339_MOESM2_ESM.pdf]
